# Ethanol withdrawal-induced adaptations in prefrontal corticotropin releasing factor receptor 1-expressing neurons regulate anxiety and conditioned rewarding effects of ethanol

**DOI:** 10.1038/s41380-022-01642-3

**Published:** 2022-06-06

**Authors:** Reesha R. Patel, Sarah A. Wolfe, Vittoria Borgonetti, Pauravi J. Gandhi, Larry Rodriguez, Angela E. Snyder, Shannon D’Ambrosio, Michal Bajo, Alain Domissy, Steven Head, Candice Contet, R. Dayne Mayfield, Amanda J. Roberts, Marisa Roberto

**Affiliations:** 1 The Scripps Research Institute, 10550N. Torrey Pines Rd, La Jolla, CA 92037 USA; 2 Dipartimento di Neuroscienze, Psicologia, Area del Farmaco e Salute del Bambino, Università degli Studi di Firenze, 50139 Firenze (FI), Italy; 3 Department of Neuroscience, The University of Texas at Austin, Austin, TX 78712 USA; 4 Waggoner Center for Alcohol and Addiction Research, The University of Texas at Austin, Austin, TX 78712 USA

**Keywords:** Neuroscience, Cell biology

## Abstract

Prefrontal circuits are thought to underlie aberrant emotion contributing to relapse in abstinence; however, the discrete cell-types and mechanisms remain largely unknown. Corticotropin-releasing factor and its cognate type-1 receptor, a prominent brain stress system, is implicated in anxiety and alcohol use disorder (AUD). Here, we tested the hypothesis that medial prefrontal cortex CRF1-expressing (mPFC^CRF1+^) neurons comprise a distinct population that exhibits neuroadaptations following withdrawal from chronic ethanol underlying AUD-related behavior. We found that mPFC^CRF1+^ neurons comprise a glutamatergic population with distinct electrophysiological properties and regulate anxiety and conditioned rewarding effects of ethanol. Notably, mPFC^CRF1+^ neurons undergo unique neuroadaptations compared to neighboring neurons including a remarkable decrease in excitability and glutamatergic signaling selectively in withdrawal, which is driven in part by the basolateral amygdala. To gain mechanistic insight into these electrophysiological adaptations, we sequenced the transcriptome of mPFC^CRF1+^ neurons and found that withdrawal leads to an increase in colony-stimulating factor 1 (CSF1) in this population. We found that selective overexpression of CSF1 in mPFC^CRF1+^ neurons is sufficient to decrease glutamate transmission, heighten anxiety, and abolish ethanol reinforcement, providing mechanistic insight into the observed mPFC^CRF1+^ synaptic adaptations in withdrawal that drive these behavioral phenotypes. Together, these findings highlight mPFC^CRF1+^ neurons as a critical site of enduring adaptations that may contribute to the persistent vulnerability to ethanol misuse in abstinence, and CSF1 as a novel target for therapeutic intervention for withdrawal-related negative affect.

## Introduction

Alcohol use disorder (AUD) is characterized by the loss of control over ethanol intake, negative emotional states in the absence of ethanol, and a compulsion to seek and consume ethanol, which is thought to heavily involve the prefrontal cortex. Individuals with an AUD have reduced prefrontal cortex volumes [1–5], and hypo-functionality of the medial prefrontal cortex (mPFC) contributes to a loss of control over limiting intake in humans with an AUD [6]. Preclinical studies also implicate the mPFC in anxiety-like behaviors and excessive ethanol drinking [7, 8]. Identifying chronic ethanol-induced adaptations that persist into withdrawal and drive aberrant behavior will provide insight into neuronal mechanisms for more efficacious therapeutic intervention, which are currently limited for AUD.

Ethanol activates neuroendocrine stress systems, which become dysregulated following chronic exposure contributing to AUD [9, 10]. Corticotropin-releasing factor (CRF) is a major brain stress signal and plays a critical role in AUD [11–13]. CRF binds the cognate CRF receptor 1 (CRF1), CRF receptor 2 (CRF2), as well as CRF binding protein (CRF-BP) [14]. While the CRF system has been extensively studied in limbic brain regions, much less is known about its functional role in prefrontal circuits. The CRF-CRF1 system is prominently expressed in the mPFC [15, 16], while CRF2 is limited [17, 18]. Specifically, CRF and CRF1 are predominantly expressed in GABAergic and glutamatergic neurons, respectively [19–21]. Functionally, injection of CRF or CRF1 antagonists in the mPFC is anxiogenic and anxiolytic, respectively [22–25], deletion of forebrain CRF1 reduces anxiety [26], mPFC CRF-CRF1 underlies stress-induced executive dysfunction [27, 28], mPFC CRF signaling impairs working memory [29, 30], and mPFC CRF interneurons regulate behavioral selection during stress [31]. Notably, withdrawal from chronic alcohol recruits mPFC GABAergic CRF neurons [32], and binge alcohol consumption decreases mPFC CRF-BP [33], which is also predominantly expressed in mPFC GABAergic neurons [34]. Taken together, CRF1-expressing mPFC neurons (mPFC^CRF1+^) have the molecular components to respond to neurochemical stress signals, that are vulnerable to repeated ethanol exposure, and are also wired to mediate anxiety-like behaviors; therefore, mPFC^CRF1+^ neurons are poised to promote relapse to ethanol use during abstinence.

Here, we tested the hypothesis that mPFC^CRF1+^ neurons comprise a distinct population that undergoes specific neuroadaptations induced by chronic ethanol and withdrawal that underlie aberrant emotional processing and ethanol drinking. Indeed, we found that mPFC^CRF1+^ neurons display reduced excitability, as well as glutamate transmission, mediated partly by basolateral amygdala (BLA) afferents. Moreover, we identified a neuroimmune mechanism, via colony-stimulating factor 1 (CSF1), underlying the observed mPFC^CRF1+^ adaptations in glutamate transmission and sufficient to induce aberrant anxiety-like behavior, which may increase relapse susceptibility. These data highlight the potential of the neuroimmune mediator, CSF1, as a promising, novel target of therapeutic intervention for AUD.

## Methods and materials

For full details see Supplementary Materials.

### Animals

Adult (>10 weeks old) male and female CRF1:GFP and CRF1:Cre mice were used [35–38]. Sample sizes for each experiment are listed in Supplementary Table 1. All procedures were approved by Scripps Institutional Animal Care and Use Committee and were consistent with the National Institutes of Health Guide for the Care and Use of Laboratory Animals.

### Chronic intermittent ethanol inhalation

To induce ethanol dependence, male mice were exposed to chronic intermittent ethanol (CIE) inhalation as previously described [37]. The average blood ethanol level achieved during CIE was 183.5 mg/dl. Cages were randomly assigned to naïve-control, dependent, and withdrawal groups.

### In situ hybridization

In situ hybridization, image acquisition, and quantification were performed as previously described [35, 39]. The following probes from ACD Biotechne were used: negative control (320751), *Crhr1* (418011), *Gad2* (439371), and *Slc17a7* (416631). Note, negligible *Crhr1* fluorescence was observed in the mouse septum (see Supplementary Fig. 1; modified from Wolfe et al. [35]), a region of low CRF1 expression [18], confirming the validity of the *Crhr1* signal.

### Immunohistochemistry

Immunohistochemistry, imaging, and quantification were performed as previously described [40]. Primary antibodies included rabbit anti-CSF1 (ab233387; Abcam), rabbit anti-Cre (69050; Novagen), or chicken anti-eGFP (ab13970; Abcam). Secondary antibodies included Alexa Fluor 488 donkey anti-rabbit (Jackson ImmunoResearch, 711-545-152), Alexa Fluor 555 donkey anti-rabbit (A32794, Invitrogen), or Alexa Fluor 488 donkey anti-chicken (703-545-155, Jackson ImmunoResearch).

### Whole-cell patch-clamp electrophysiology and optogenetics

Whole-cell voltage-clamp and current-clamp recordings were collected, in an unblinded fashion, from pyramidal neurons, morphologically confirmed visually and with a cell capacitance criterion of >80 pF, and analyzed as previously described [40, 41]. A K-gluconate internal solution was used to record spontaneous excitatory postsynaptic currents (sEPSCs) in artificial cerebrospinal fluid (ACSF), miniature excitatory postsynaptic currents (mEPSCs) in the presence of 30 µM bicuculline (BIC; Tocris) and 0.5 µM tetrodotoxin (TTX; Sigma Aldrich), and excitability in ACSF.

For optogenetic experiments, channelrhodopsin-2 (ChR2)-photocurrents were measured using wide-field illumination. Mono-synaptic connectivity was measured in ACSF containing 30 µM BIC, 0.5 µM TTX, and 100 µM 4-aminopyridine (4-AP). AMPA and NMDA currents were recorded from a holding potential of −80 mV and +40 mV, respectively, using an Cs-methanesulfonate internal solution. Data analysis was conducted in a blind fashion until the final step of grouping cells and statistics.

### Site-specific viral injection surgery

Viral injections were performed as previously described [42]. Cages were randomly assigned into control and treatment groups for each experiment. For optogenetics AAV2-hSyn-ChR2-mCherry was injected into the BLA (AP: −1.5, DV: −4.9, ML: ± 3.4; UNC Vector Core), for mPFC^CRF1+^ ablation AAV2-flex-taCasp3-TEVp (UNC Vector Core), for mPFC^CRF1+^ CSF1 overexpression AAV2-CMV-DIO-mCSF1-2A-mCherry (Vector Biolabs), or control AAV2-hSyn-DIO-mCherry (Addgene) was injected into the mPFC (AP: + 1.9, DV: −2.4, ML: ± 0.5).

### Behavioral testing

Novelty-induced suppression of feeding was performed as previously described [42]. Ethanol place conditioning was performed in a two-chamber apparatus consisting of three phases: 1-day pre-conditioning, 12-days of conditioning, and 1-day test. During conditioning, mice received injections of 2 g/kg ethanol or saline and were confined to one compartment. During pre-conditioning and test, mice were given access to the entire chamber. The time spent in each compartment was measured. Experimenters were blinded to groups during testing.

### Fluorescence activated cell sorting and RNA sequencing

The mPFC from CRF1:GFP mice was microdissected, tissue was dissociated and sorted, and RNA was isolated from sorted cells as previously described [43]. Samples were used for RNA sequencing and mapped to the mouse genome. Differential gene expression was assessed [44]. Advaita bioinformatics was used for gene ontology, pathway, and network analysis. Data will be available on Gene Expression Omnibus (accession number GSE202936).

### Data statistics

Data are presented as mean ± standard error (SEM) with individual data points overlayed, and *N and n* represents sample number of mice and cells, respectively. Sample sizes were choosen based on previously conducted experiments. Grubb’s outlier test was used to identify outliers, which were excluded from datasets. All statistical tests, stated in the figure legend for each experiment, met the appropriate assumptions regarding normal distribution and homoscedasticity of data, were two-tailed, and *p*-values were adjusted for multiple comparisons as appropriate. A *p*-value of <0.05 was considered the cutoff for significance.

## Results

### mPFC^CRF1+^ neurons comprise a distinct glutamatergic population that regulates anxiety and ethanol reinforcement

**Fig. 1:**
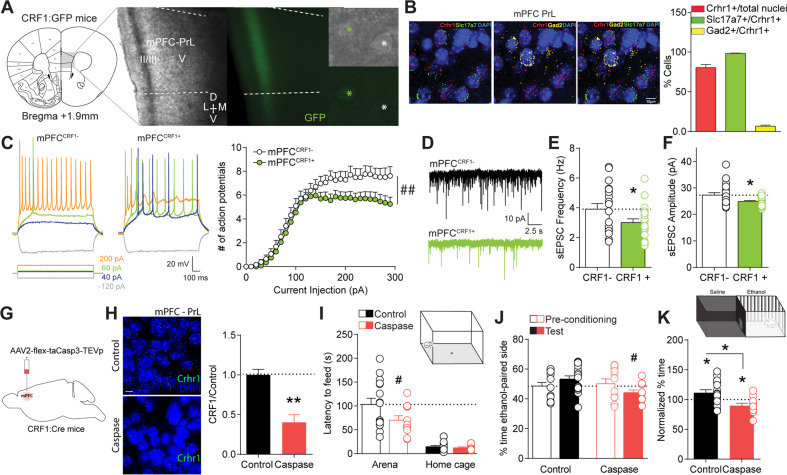
mPFC^CRF1+^ neurons comprise a distinct glutamatergic population that regulates anxiety and conditioned rewarding effects of ethanol. **A** Coronal mouse brain atlas [80] image of medial prefrontal cortex (mPFC), corresponding 4X magnification, brightfield and GFP fluorescence images of boxed region depicting the mPFC and 40X magnification, brightfield and GFP fluorescence images depicting neighboring GFP positive (green asterisk; mPFC^CRF1+^) and negative (white asterisk; mPFC^CRF1−^) mPFC prelimbic (PrL) neurons. **B** Representative in situ hybridization images of colocalization of Crhr1 (red), Slc17a7 (green), Gad2 (yellow), and DAPI (blue) in the mPFC PrL layer 2/3 subregion and summary graph of percent nuclei expressing. Scale bar = 10 µM. 4 images from *N* = 2 male mice. **C** Representative voltage traces of action potential firing elicited by increasing current injections (−120, 40, 60 and 200 pA, 1 s) in mPFC PrL CRF1 non-expressing (mPFC^CRF1−^) and CRF1 expressing (mPFC^CRF1+^) pyramidal neurons. Average number of action potentials elicited by increasing current injections in mPFC^CRF1−^ and mPFC^CRF1+^ neurons with significant effects of current injection F(29,1972) = 87.27, *p* < 0.0001; group F(1,68) = 4.26, *p* = 0.04; and interaction between current injection and group F(29, 1972) = 1.99, *p* = 0.001 by two-way ANOVA from *n* = 31–39 cells and *N* = 10 male mice. **D** Representative traces of spontaneous excitatory post-synaptic currents (sEPSCs) recorded with a hold potential of −70 mV. **E**, **F** Average sEPSC frequency and amplitude in mPFC^CRF1−^ (black) and mPFC^CRF1+^ (green) neurons. *n* = 19–21 cells from *N* = 8 male mice; *p < 0.05 by t-test. **G** Schematic of viral strategy for caspase-mediated ablation of mPFC CRF1-expressing (mPFC^CRF1+^) neurons in a Cre-dependent manner in CRF1:Cre mice (Caspase). **H** Representative in situ hybridization images depicting Crhr1 (green) and DAPI (blue) expression in the mPFC of control (top) and caspase (bottom) mice. Quantification of Crhr1 positive nuclei in mPFC PrL layer 2/3 of control and caspase mice relative to control group. *N* = 3 male mice/group; **p < 0.01 by *t*-test. Scale bar = 10 µM. **I** Latency to feed in an open arena (open bars) and home cage (filled bars) in novelty-suppressed feeding test in control (black) and mPFC^CRF1+^ ablated (red) mice with significant main effects of condition F(1,23) = 70.78, *p* < 0.0001 and group F(1,23) = 4.66, *p* = 0.04 by two-way ANOVA, and post hoc significance ^#^*p* < 0.05 represented in panel. **J** Percent time spent in the ethanol paired context during the pre-conditioning phase (open bars) and following ethanol conditioning (filled bars) in the ethanol place conditioning test in control (black) and mPFC^CRF1+^ ablated (red) mice with a significant interaction effect F(1,24) = 11.05, *p* = 0.002 by two-way ANOVA, and post hoc significance ^#^*p* < 0.05 represented in panel. **K** Pre-conditioning normalized percent time spent in the ethanol-paired side with significant *p < 0.05 effects by one-sample t-test and unpaired t-test. *N* = 11–15 male mice.

To determine the identity and electrophysiological properties of mPFC^CRF1+^ neurons, we used male, CRF1:GFP reporter mice for in situ hybridization and ex vivo whole-cell patch-clamp electrophysiology. mPFC^CRF1+^ neurons are densely distributed in layer 2/3 of the prelimbic (PrL) subdivision (Fig. 1A). Nuclei expressing *Crhr1* mRNA predominantly co-express *Slc17a7* compared to *Gad2* mRNA, suggesting that mPFC^CRF1+^ neurons are primarily glutamatergic (Fig. 1B). mPFC^CRF1+^ pyramidal neurons showed a distinct electrophysiological signature compared to neighboring mPFC PrL CRF1 non-expressing pyramidal neurons (mPFC^CRF1−^) including reduced excitability, less voltage sag, greater initial action potential amplitude, and a more depolarized resting membrane potential (Fig. 1C, Supplementary Fig. 2). Moreover, mPFC^CRF1+^ neurons had reduced spontaneous excitatory postsynaptic current (sEPSC) frequency and amplitude, suggesting less glutamatergic input and post-synaptic transmission in this population (Fig. 1D–F). No effect on sEPSC kinetics were observed (data not shown). In addition, we found that acute CRF application (200 nM) decreases excitability of mPFC^CRF1+^ and mPFC^CRF1−^ neurons (Supplementary Fig. 3 and Supplementary Table 3). These data suggest that mPFC^CRF1+^ neurons comprise a unique glutamatergic population with distinct electrophysiological properties.

To assess the behavioral relevance of this population, we selectively ablated mPFC^CRF1+^ neurons in male, CRF1:Cre mice and examined the behavioral consequence (Fig. 1G–K, Supplementary Fig. 4). We measured anxiety-like behavior using the novelty suppressed feeding test [45]. Ablation of mPFC^CRF1+^ neurons decreased latency to feed in the arena, suggesting a decrease in anxiety-like behavior (Fig. 1I). No difference in latency to feed in the home cage was observed, suggesting a similar motivation for food. We also measured ethanol place conditioning to assess conditioned ethanol reward. Control mice preferred the ethanol-associated context, highlighting the conditioned reinforcing properties of ethanol (Fig. 1J, K). Remarkably, ablation of mPFC^CRF1+^ neurons induced a conditioned place aversion to the ethanol-associated context, suggesting conditioned aversion to ethanol. Together, these findings demonstrate that mPFC^CRF1+^ neurons regulate anxiety-like behaviors and conditioned rewarding effects of ethanol, supporting the potential role of this population in aberrant emotional processing in abstinence.

### Withdrawal selectively decreases mPFC^CRF1+^ excitability

**Fig. 2:**
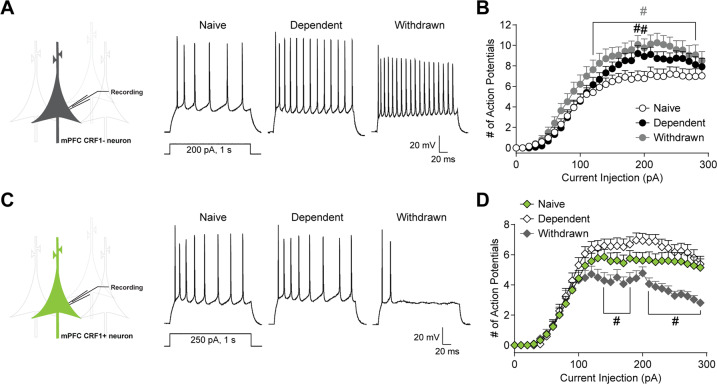
Withdrawal selectively decreases mPFC^CRF1+^ excitability. **A** Representative voltage traces of action potential firing elicited by a 200 pA current injection in mPFC CRF1 non-expressing (mPFC^CRF1−^) pyramidal neurons from naïve, dependent and withdrawn mice. **B** Average number of action potentials elicited by increasing current injections in mPFC^CRF1−^ neurons from naïve (white circles), dependent (black circles), and withdrawn (grey circles) mice with main effects of current injection F(29,2320) = 111.9, *p* < 0.0001; group F(2,80) = 5.22, *p* = 0.007; and a trend toward an interaction effect F(58,2320) = 1.32, *p* = 0.05 by two-way ANOVA from *n* = 24–34 cells and *N* = 6–9 male mice, and post hoc significance ^#^*p* < 0.05 compared to naive is represented in panel. **C** Representative voltage traces of action potential firing elicited by a 250 pA, 1 s current injection in mPFC CRF1-expressing (mPFC^CRF1+^) pyramidal neurons from naïve, dependent, and withdrawn mice. **D** Average number of action potentials elicited by increasing current injections in mPFC^CRF1+^ neurons from naïve (green diamonds), dependent (white diamonds), and withdrawn (grey diamonds) mice with main effects for current injection F(29,2842) = 139.2, *p* < 0.0001; group F(2,98) = 5.60, *p* = 0.005; and interaction effect F(58,2842) = 4.00, *p* < 0.001 by two-way ANOVA from *n* = 29–40 cells and *N* = 6–9 male mice, and post hoc significance ^#^*p* < 0.05 compared to naive is represented in panel.

Since mPFC^CRF1+^ neurons regulate negative affective behaviors and reinforcing properties of ethanol that can contribute to relapse, we asked if this population undergoes specific neuroadaptations following withdrawal from chronic ethanol. We exposed male CRF1:GFP mice to chronic intermittent ethanol (CIE) inhalation to generate dependent and withdrawn mice that were 5–8 days into forced abstinence. We found that mPFC^CRF1−^ pyramidal neuron excitability is increased in dependent and withdrawn mice (Fig. 2A, B). Electrophysiological properties are summarized in Supplementary Table 2. Overall, mPFC^CRF1−^ neuronal excitability is increased by ethanol dependence and withdrawal.

We then selectively recorded from neighboring mPFC^CRF1+^ pyramidal neurons and found no significant effect of ethanol dependence on neuronal excitability (Fig. 2C, D). Notably, withdrawal markedly decreased mPFC^CRF1+^ excitability compared to naïve, pointing to the selective sensitivity of mPFC^CRF1+^ neurons to withdrawal. Interestingly, there was a significant reduction in initial action potential amplitude in mPFC^CRF1+^ neurons during withdrawal, suggesting ion channel expression and/or kinetics may partly underlie the observed decreased excitability (Supplementary Table 2, Fig. 4). These data suggest that mPFC^CRF1+^ neurons are particularly sensitive to withdrawal from chronic ethanol and undergo distinct neuroadaptations in excitability in response to ethanol withdrawal.

### Withdrawal selectively decreases post-synaptic glutamate transmission in mPFC^CRF1+^ neurons, which is partly mediated by the basolateral amygdala

**Fig. 3:**
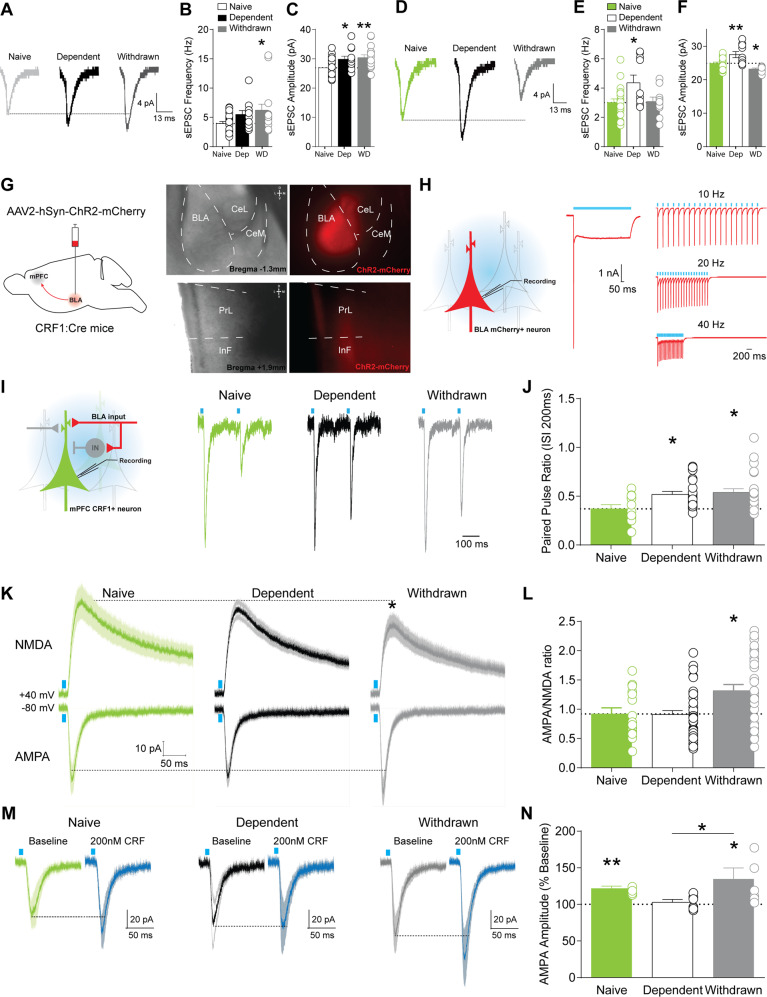
Withdrawal selectively decreases post-synaptic glutamate transmission in mPFC^CRF1+^ neurons, which is partly mediated by the basolateral amygdala. **A** Representative traces of average spontaneous excitatory post-synaptic currents (sEPSC) recorded with a holding potential of −70 mV in mPFC^CRF1−^ pyramidal neurons from naïve, dependent, and withdrawn mice. **B**, **C** Average sEPSC frequency and amplitude in mPFC^CRF1−^ neurons from naïve (white), dependent (black), and withdrawn (grey) mice. *n* = 12–22 cells from *N* = 6–10 male mice; *p < 0.05, **p < 0.01 by one-way ANOVA and post hoc multiple comparisons compared to naive. **D** Representative traces of average sEPSCs recorded with a holding potential of −70 mV in mPFC^CRF1+^ neurons from naïve, dependent, and withdrawn mice. **E**, **F** Average sEPSC frequency and amplitude in mPFC^CRF1+^ pyramidal neurons from naïve (green), dependent (white), and withdrawn (grey) mice. *n* = 10–21 cells from *N* = 6–10 male mice; *p < 0.05, **p < 0.01 by one-way ANOVA and post hoc multiple comparisons compared to naive. **G** Schematic of viral strategy for ex vivo optogenetic circuit dissection of glutamate transmission in the basolateral amygdala (BLA) to mPFC^CRF1+^ pathway. Representative 4X magnification, brightfield and mCherry fluorescence images of injection site expression of channelrhodopsin-2 (ChR2)-mCherry in the BLA (right, top) and corresponding ChR2-mCherry BLA terminal in the mPFC (right, bottom). **H** ChR2-mediated photocurrents in mCherry expressing BLA neurons elicited by pulses and increasing trains of blue-light stimulation from a holding potential of −70 mV. **I** Representative BLA-mediated AMPA currents in mPFC^CRF1+^ neurons elicited by paired pulse stimulation (traces scaled to first EPSC) of two consecutive blue light pulses (3 ms, 470 nm) with an interstimulus interval of 200 ms from a holding potential −80 mV. **J** Average paired pulse ratio (i.e., amplitude of the second response normalized to that of the first) in mPFC^CRF1+^ neurons from naïve, dependent, and withdrawn mice with a significant F(2,57) = 4.27, *p* = 0.01 effect by one-way ANOVA, and post hoc significance *p < 0.05 compared to naïve is represented in *p*anel. *n* = 12–27 cells from *N* = 5–6 male mice. **K** Average ± SEM BLA-mediated NMDA (top row) and AMPA (bottom row) currents in mPFC^CRF1+^ neurons from naïve (green), dependent (black), and withdrawn (grey) mice. **L** Average AMPA/NMDA ratio in mPFC^CRF1+^ neurons from naïve, dependent, and withdrawn mice with a significant F(2,86) = 6.79, *p* = 0.001 effect by one-way ANOVA, and post hoc significance *p < 0.05 compared to naïve is represented in panel. *n* = 17–39 cells from *N* = 5–6 male mice. **M** Average ± SEM BLA-mediated AMPA currents elicited by blue light pulses during baseline and following 200 nM CRF application from a holding potential of −80 mV in mPFC^CRF1+^ neurons from naive, dependent, and withdrawn mice. **N** Average baseline normalized AMPA amplitude elicited by optical stimulation of BLA terminals with a significant F(2,16) = 3.55, *p* = 0.05 effect of CRF across groups by one-way ANOVA and post hoc multiple comparisons, and significant *p < 0.05 and **p < 0.01 effects of CRF by one sample *t*-test. *n* = 4–11 cells from *N* = 3–6 male mice per group.

Given that CRF signaling remodels spine density contributing to anxiety-like behaviors [46, 47], we next asked whether chronic ethanol alters glutamatergic singling in mPFC^CRF1+^ pyramidal neurons in male CRF1:GFP mice. We found that mPFC^CRF1−^ neurons displayed a trend toward and a significant increase in sEPSCs frequency in dependent and withdrawn mice, respectively, indicative of increased presynaptic glutamate release (Fig. 3A, B). mPFC^CRF1−^ neurons also displayed increased sEPSC amplitude and current kinetics in dependent as well as withdrawn mice, suggesting enhanced postsynaptic glutamatergic signaling (Fig. 3C, Supplementary Fig. 5). Similar to mPFC^CRF1−^ neurons, dependence increased sEPSC frequency, amplitude, and kinetics in mPFC^CRF1+^ pyramidal neurons compared to naïve (Fig. 3D–F, Supplementary Fig. 5). However, the increase in sEPSC frequency was lost in withdrawn mice and significant decreases in sEPSC amplitude and current kinetics were observed, suggesting a decrease in postsynaptic glutamate transmission in mPFC^CRF1+^ neurons in withdrawal. Together, these findings further highlight the unique sensitivity of mPFC^CRF1+^ neurons to withdrawal and point to reduced glutamatergic signaling in this population as a potential mechanism underlying heightened anxiety-like behavior in withdrawal.

The BLA and mPFC show excitatory coupling and enhanced activity during anxiety, and activation of BLA inputs to the mPFC induces anxiety-like behaviors [48, 49], suggesting the BLA-mPFC circuit is a candidate in underlying aberrant emotional processing following withdrawal. To measure BLA-mPFC^CRF1+^ connectivity, we expressed ChR2-mCherry in the BLA of CRF:GFP mice (Fig. 3G) and measured light-evoked post-synaptic potentials in mPFC^CRF1+^ neurons. Blue light reliably elicited time-locked photocurrents in mCherry-expressing BLA neurons, demonstrating precise control of BLA neuronal activity (Fig. 3H). To assess presynaptic BLA-mediated glutamate release onto mPFC^CRF1+^ neurons, we measured responses in mPFC^CRF1+^ neurons to optical paired pulse stimulation of BLA terminals in the mPFC (Fig. 3I, J). The paired pulse ratio was significantly increased in dependent and withdrawn mice, suggesting a decrease in glutamate release onto mPFC^CRF1+^ neurons following chronic ethanol. To assess postsynaptic alterations in BLA-mPFC^CRF1+^ connectivity, we measured optically evoked mono-synaptic AMPA and NMDA currents in mPFC^CRF1+^ neurons (Fig. 3K, L). NMDA amplitude was significantly decreased, and correspondingly the AMPA/NMDA ratio was significantly increased in withdrawn mice compared to naïve, indicating a selective decrease in postsynaptic glutamate transmission in the BLA-mPFC^CRF1+^ pathway in withdrawn mice. Together, these findings demonstrate that the upstream BLA, which directly innervates mPFC^CRF1+^ neurons, is dysregulated by dependence and that this dysregulation persists into withdrawal. Dysregulation of the BLA temporally precedes the selective dysregulation of mPFC^CRF1+^ neurons in withdrawal, suggesting that the BLA partly drives aberrant activity of mPFC^CRF1+^ neurons in withdrawal.

Since CRF-CRF1 signaling can potently modulate neuronal activity and synaptic plasticity [11, 50, 51], we also examined the impact of CRF on the BLA-mPFC^CRF1+^ pathway. CRF (200 nM) potentiated BLA-mediated AMPA currents in mPFC^CRF1+^ neurons in naïve mice (Fig. 3M, N), which was abolished by dependence, but recovers in withdrawal, suggesting a transient neuroadaptation to CRF signaling in mPFC^CRF1+^ neurons. CRF did not significantly alter BLA-mediated NMDA currents or the paired pulse ratio (Supplementary Fig. 6). Of note, CRF does not significantly alter global miniature EPSCs (mEPSCs) in mPFC^CRF1+^ neurons from naïve or dependent mice, but selectively potentiates mEPSCs in withdrawal (Supplementary Fig. 7). Together these findings demonstrate that CRF can potentiate BLA-mPFC^CRF1+^ connectivity.

### Withdrawal from chronic ethanol alters the transcriptome of mPFC^CRF1+^ neurons – upregulating CSF1

**Fig. 4:**
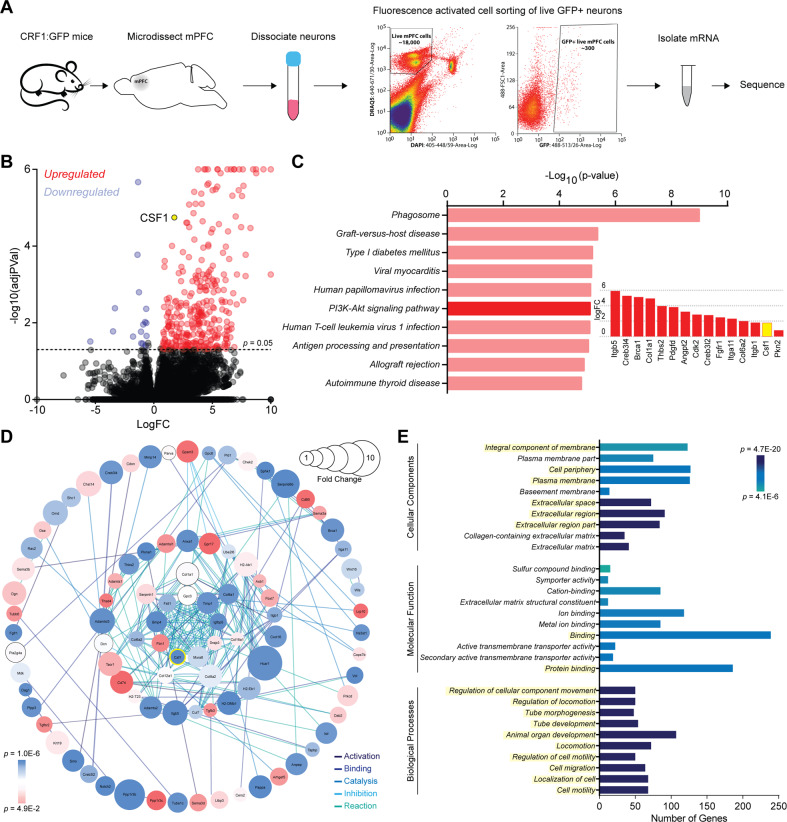
Withdrawal induces whole transcriptomic changes in mPFC^CRF1+^ neurons. **A** Schematic of isolation of mPFC^CRF1+^ neurons using fluorescence activated cell sorting from naïve and withdrawn mice for whole transcriptomic analysis. **B** Volcano plot of average log fold change plotted against the log of the adjusted p-value for expressed genes. Significantly (*p* < 0.05) upregulated and downregulated genes in withdrawn mice compared to naive are represented in red and blue, respectively. *N* = 5–6 male mice. **C** Top ten most significantly impacted pathways based on the differentially expressed genes (DEGs). Log fold change in the DEGs included in the PI3K-AKT signaling pathway where CSF1 is highlighted in yellow (inset). **D** Network analysis depicting interactions between DEGs. **E** Top ten gene ontology (GO) terms overrepresented among DEGs in three domains including: biological processes, molecular function, and cellular components. CSF1 is included in yellow highlighted GO terms.

To gain mechanistic insight into the observed electrophysiological neuroadaptations, we sequenced the transcriptome of mPFC^CRF1+^ neurons from naïve and withdrawn mice. Using male CRF1:GFP mice, we sequenced RNA from mPFC^CRF1+^ neurons isolated using fluorescence-activated cell sorting (Fig. 4A). We detected over forty-thousand genes and found 344 significant differentially expressed genes (DEGs) (Fig. 4B). We then identified pathways, hub genes, and functional processes most impacted by withdrawal from chronic ethanol in mPFC^CRF1+^ neurons (Fig. 4C-E). From these analyses and a literature review, we identified an intriguing candidate, colony stimulating factor 1 (CSF1) for further in-depth analysis. CSF1 is highlighted in yellow throughout Fig. 4. We first identified the most significantly impacted pathways based on the DEGs (Fig. 4, Supplementary Table 4). CSF1 is included in the gene list comprising the PI3K-Akt signaling pathway, which was one of the top ten impacted pathways (Fig. 4C***inset***). In addition, several other signaling, peptide, and immune pathways were identified along with a specific alcoholism pathway, highlighting a central role of mPFC^CRF1+^ neurons in an AUD phenotype (Supplementary Table 4). Network analysis of DEGs revealed the known interactions and relationships between genes (Fig. 4D). Notably, CSF1 is a hub gene, indicated by its centrality in the network showing a high degree of connectivity among the DEGs. Lastly, to identify the functional processes in mPFC^CRF1+^ neurons that are most impacted by withdrawal from chronic ethanol, we used gene ontology (GO) analysis. The top ten GO terms within the three domains including: biological processes, molecular functions, and cellular components that are significantly overrepresented in the set of DEGs were identified (Fig. 4E). CSF1 is among the top ten GO terms for biological processes and many of the GO terms for molecular components as well. These data highlight the fundamental neurobiological adaptations induced by withdrawal from chronic ethanol in mPFC^CRF1+^ neurons, and recruitment of CSF1 as a candidate gene that could underlie critical neuroadaptations driving aberrant behavior.

### CSF1 overexpression in mPFC^CRF1+^ decreases post-synaptic glutamate transmission and is sufficient to increase anxiety-like behavior while abolishing conditioned ethanol reward

**Fig. 5:**
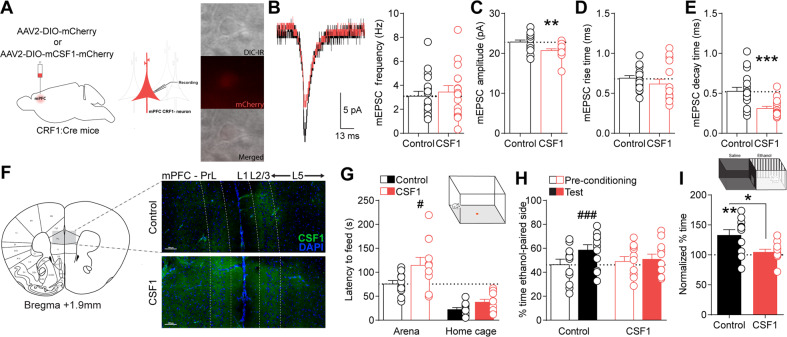
CSF1 overexpression in mPFC^CRF1+^ neurons decreases post-synaptic glutamate transmission and is sufficient to increase anxiety-like behavior and abolish ethanol place preference. **A** Schematic of viral strategy to selectively overexpress CSF1 in a Cre-dependent manner in mPFC^CRF1+^ neurons using ethanol-naïve CRF1:Cre mice. 40X magnification for brightfield, mCherry fluorescence, and merged images of a CSF1-mCherry expressing mPFC^CRF1+^ neuron. **B** Representative current trace of average mEPSC in control mCherry (black) and CSF1-mCherry (red) expressing mPFC^CRF1+^ neuron. Average mEPSC frequency in control mCherry and CSF1-mCherry expressing mPFC^CRF1+^ pyramidal neurons. **C**–**E** Average mEPSC amplitude, rise time, and decay time in control mCherry and CSF1-mCherry expressing mPFC^CRF1+^ neurons. *n* = 16–18 cells from *N* = 4 female mice; **p < 0.01, ***p < 0.001 by *t*-test. **F** Coronal mouse brain atlas image of mPFC and corresponding representative 10X magnification fluorescence image of boxed region depicting CSF1 expression in CSF1-overexpressing compared to control mice. Scale bar = 100 µM. **G** Latency to feed in an open arena (open bars) and home cage (filled bars) in the novelty-suppressed feeding test in control (black) and mPFC^CRF1+^ CSF1 overexpressing (red) mice with significant main effects of condition F(1,19) = 58.59, *p* < 0.001 and group F(1,19) = 5.67, *p* = 0.02 by two-way ANOVA, and post hoc significance ^#^*p* < 0.05 is represented in panel^.^ **H** Percent time spent in the ethanol paired context during the pre-conditioning phase (open bars) and following ethanol conditioning (filled bars) in the ethanol place conditioning test for control (black) and mPFC^CRF1+^ CSF1 overexpressing (red) mice with a significant main effect of condition F(1,20) = 11.43, *p* = 0.003 and interaction effect F(1,20) = 6.41, *p* = 0.01 by two-way ANOVA, and post hoc significance ^###^*p* < 0.001 is represented in panel. **I** Pre-conditioning normalized percent time spent in the ethanol-paired side with significant **p < 0.01 effects by one-sample t-test and *p < 0.05 unpaired *t*-test. *N* = 11 female mice/group.

Notably, CSF1 is upregulated by mPFC neurons following stress leading to microglia-mediated spine pruning underlying anxiety- and depressive-like behaviors [52]. Therefore, we hypothesized that upregulation of CSF1, as revealed by our transcriptomic analysis, may be a mechanism underlying the reduced post-synaptic glutamate transmission observed selectively in mPFC^CRF1+^ pyramidal neurons following withdrawal (Fig. 3D; Supplementary Fig. 5). To test this, we selectively overexpressed CSF1 in mPFC^CRF1+^ neurons, using female CRF1:Cre mice, and used whole-cell patch-clamp electrophysiology to assess mEPSCs (Fig. 5A; Supplementary Fig. 8). We found that CSF1 overexpression in mPFC^CRF1+^ neurons decreased mEPSC amplitude and decay kinetics compared to control but did not alter mEPSC frequency or rise kinetics (Fig. 5B–E). CSF1 overexpression in mPFC^CRF1+^ neurons did not significantly alter excitability of mPFC^CRF1+^ or mPFC^CRF1−^ neurons (Supplementary Table 5; Supplementary Fig. 9). These data show that CSF1 overexpression in mPFC^CRF1+^ neurons reduce post-synaptic glutamate transmission, identifying a potential mechanism underlying the reduced glutamatergic signaling selectively observed in this population following withdrawal.

We then asked if CSF1 overexpression in mPFC^CRF1+^ neurons is sufficient to alter behavior (Fig. 5F–I; Supplementary Fig. 10). Immunohistochemical staining confirmed mPFC CSF1 overexpression in CSF1-mCherry compared to mCherry control mice (Fig. 5F). We found that CSF1 overexpression significantly increased the latency to feed in an open arena in the novelty-suppressed feeding test, suggesting an increase in anxiety-like behavior (Fig. 5G). CSF1 overexpression in mPFC^CRF1+^ neurons abolished ethanol place preference, suggesting a loss in the reinforcing properties of ethanol (Fig. 5H, I). These findings demonstrate that CSF1 overexpression in mPFC^CRF1+^ neurons is sufficient to induce anxiety-like behaviors and abolish the rewarding properties of ethanol, providing mechanistic insight into the regulation of these aberrant behaviors following ethanol withdrawal.

## Discussion

This study pinpoints behaviorally relevant molecular-, cell-type-, and circuit- specific adaptations selectively induced by withdrawal from chronic ethanol which underlie negative affective behavior and conditioned ethanol reward. We found that mPFC^CRF1+^ neurons display decreased excitability and glutamatergic transmission in withdrawal, suggesting that they comprise a distinct population highly vulnerable to ethanol withdrawal. Moreover, we found that the BLA in part drives the downstream dysregulation of mPFC^CRF1+^ neurons in withdrawal. Ablation of mPFC^CRF1+^ neurons decreased anxiety-like behavior and induced conditioned aversion to ethanol, supporting a unique role of mPFC^CRF1+^ neurons in AUD-related behaviors. Notably, we found that ethanol withdrawal fundamentally alters the neurobiology of mPFC^CRF1+^ neurons including overexpression of colony stimulating factor 1. Selective overexpression of CSF1 in mPFC^CRF1+^ neurons was sufficient to decrease postsynaptic glutamate transmission and induce behavioral deficits in anxiety and conditioned ethanol reward, providing mechanistic insight into the observed withdrawal-associated neuroadaptations in glutamate transmission and aberrant behavior. Taken together, we have identified a distinct mPFC CRF1-expressing subpopulation in the BLA-mPFC circuit that undergoes specific neuroadaptations following ethanol withdrawal, potentially underlying increased vulnerability to relapse during abstinence.

The CRF-CRF1 system is prominently expressed in the mPFC and serves as a potent regulator of neuronal activity, structural and functional plasticity, and emotional and cognitive behaviors. Stress-induced CRF-CRF1 signaling results in mPFC and hippocampal dendritic atrophy underlying anxiety and memory deficits [46, 47]. Consistent with CRF’s role in synaptic remodeling [53], we found that mPFC^CRF1+^ neurons have reduced basal postsynaptic glutamatergic transmission, possibly due to ongoing basal CRF-CRF1 signaling regulating spine density. CRF also increases mPFC neuronal excitability [19, 20]. Accordingly, mPFC^CRF1+^ had a depolarized resting membrane potential, potentially due to basal CRF-CRF1 signaling induced persistent sodium or Ih current [19, 54–57]. Though, mPFC^CRF1+^ neurons exhibited overall reduced excitability, voltage sag, and glutamate transmission under basal conditions. Moreover, CRF modulates mPFC glutamate transmission. We found that CRF enhances BLA-mPFC^CRF1+^ connectivity. Notably, we didn’t observe a significant impact of CRF on global mEPSCs in mPFC^CRF1+^ neurons, suggesting that CRF may impact select circuits comprising mPFC^CRF1+^ neurons. Indeed, CRF-induced mPFC excitatory post-synaptic currents requires BLA input [58], suggesting that CRF may bias toward a greater influence of the BLA on mPFC activity. Given CRF’s neuromodulatory role, it is predictable that mPFC CRF-CRF1 signaling impacts behavior. Our findings further support a role for mPFC^CRF1+^ neurons in anxiety-like behavior and conditioned rewarding effects of ethanol, although it is possible that compenstatory changes may contribute to these behavioral effects. The pleiotropic effects of mPFC CRF-CRF1 signaling on neuronal physiology and AUD-related behaviors positions mPFC^CRF1+^ neurons to mediate aberrant behaviors underlying AUD.

While the CRF-CRF1 system has been strongly implicated in preclinical models of AUD particularly in limbic brain regions [11], considerably less is known about its role in the mPFC. Here we hypothesized that mPFC^CRF1+^ neurons are uniquely sensitive to chronic ethanol and undergo selective dysregulation underlying AUD-related behaviors. Our previous work demonstrated differential sensitivity in GABAergic signaling in CeA^CRF1+^ neurons compared to CeA^CRF1−^ neurons to acute and chronic ethanol [36, 37]. In this study, mPFC^CRF1+^ neurons exhibited decreases in excitability and glutamate transmission selectively in withdrawal. In contrast, mPFC^CRF1−^ displayed increases in excitability and glutamate transmission following dependence and withdrawal. This finding is consistent with previous work showing an increase in mPFC neuronal excitability following chronic intermittent ethanol exposure [59, 60], which may contribute to CIE-induced cognitive deficits [60–63]. This highlights the selective sensitivity of mPFC^CRF1+^ to withdrawal and its potential in uniquely contributing to AUD-related behaviors. Decreased mPFC^CRF1+^ excitability may be driven by changes in glutamatergic drive as well as in intrinsic properties. Indeed, transcriptomic analysis identified an increase in *SCN5A*, encoding the sodium channel Nav1.5, which may underlie the reduced action potential amplitude of mPFC^CRF1+^ neurons in withdrawal. Notably, withdrawal leads to the activation of mPFC CRF GABAergic neurons [32], which presumably innervate mPFC^CRF1+^ neurons and may also contribute to the reduced mPFC^CRF1+^ excitability in withdrawal, as we observed CRF decreases mPFC^CRF1+^ excitability. Further identification of the circuitry comprising mPFC^CRF1+^ neurons will provide a framework to understand the role of this population in AUD.

The BLA sends dense glutamatergic projections to the mPFC and contributes to anxiety-related behaviors and addiction [64]. We found that BLA-mPFC^CRF1+^ synapses undergo adaptations, suggesting a reduced probability of glutamate release from BLA terminals following chronic ethanol which persists into withdrawal. Post-synaptic BLA connections to mPFC^CRF1+^ neurons exclusively undergo adaptations in withdrawal, specifically a decrease in NMDA current leading to increased AMPA/NMDA ratio. The temporal sequence of these pathological synaptic adaptations suggests that the BLA partly drives the aberrant glutamatergic transmission observed in mPFC^CRF1+^ neurons following withdrawal. mPFC NMDA hypofunction is associated with cognitive impairment [65, 66] and increased opiate reward sensitivity, which is dependent on the BLA [67]. While not directly tested here, it is possible that reduced mPFC^CRF1+^ NMDA current following withdrawal may increase the rewarding properties of ethanol, given the role of mPFC^CRF1+^ neurons in conditioned ethanol reward. A global understanding of the neurobiological changes will provide insight into concurrent mechanisms driving aberrant behavior.

To examine global molecular changes associated with withdrawal selectively in mPFC^CRF1+^ neurons, we used cell-type specific transcriptomic analysis. Notably, overrepresented transcriptomic changes in this single, specific mPFC^CRF1+^ population were sufficient to identify a significant perturbation in biological pathways associated with ‘alcoholism’, supporting the role of this population in AUD. Interestingly, the PI3K-Akt pathway, a highly impacted pathway by withdrawal, has previously been identified as a mPFC molecular mechanism underlying ethanol intake and anxiety in withdrawal [68]. In addition, our bioinformatic analysis identified CSF1, a neuroimmune mediator important for the development and maintenance of microglia, as a hub gene [69]. Indeed, stress-induced increases in mPFC CSF1 expression leads to microglia-mediated synaptic pruning, and CSF1 knockdown was sufficient to reverse stress-induced anxiety and depressive behaviors [52]. We found that CSF1 overexpression in mPFC^CRF1+^ neurons was sufficient to induce decreased postsynaptic glutamate transmission in this population, supporting a microglia-mediated mechanism underlying synaptic adaptations. In line with this, withdrawal from chronic ethanol increases mPFC microglial reactivity [70, 71]. Indeed, mPFC^CRF1+^ CSF1 overexpression increases anxiety-like behavior, highlighting CSF1 overexpression as a critical neuroadaptation that may contribute to negative affect in withdrawal. Note, CSF1 overexpression also induced a loss of the conditioned rewarding effects of ethanol. While this may appear counterintuitive with the anxiogenic effects of CSF1 overexpression, this finding is consistent with a shift in negative reinforcement driven drinking, rather than the rewarding effects of ethanol itself, following chronic ethanol [11, 72, 73]. Thus, CSF1 overexpression in mPFC^CRF1+^ neurons induced by withdrawal from chronic ethanol may drive escalated drinking by increasing negative affective states, contributing to negative reinforcement mechanisms motivating drinking in AUD.

Our findings suggest that mPFC^CRF1+^ regulate anxiety-like behavior, due to the observed decreases in anxiety-like behavior following ablation of this population. These findings suggest that synaptic adaptations in mPFC^CRF1+^ neurons may contribute to heightened anxiety following withdrawal. Indeed, we demonstrated that withdrawal-induced molecular adaptations in CSF1 expression in mPFC^CRF1+^ neurons are sufficient to heighten anxiety and recapitulate the reduced postsynaptic glutamate transmission seen in this population in withdrawal. Together, mPFC^CRF1+^ neurons regulate withdrawal-related ethanol drinking and anxiety-like behaviors and undergo unique adaptations following withdrawal from chronic ethanol exposure that may increase vulnerability to relapse.

Of note, while both male and female mice were used, all electrophysiological data and mPFC^CRF1+^ ablation behavioral experiment were conducted in male mice, while CSF1 overexpression behavioral experiments were conducted in female mice. We found that mPFC^CRF1+^ ablation and CSF1 overexpression studies both altered behavior in the novelty-suppressed feeding test, suggesting this population regulates anxiety-like behavior in both male and female mice. Although, there are sex differences in mPFC circuits and anxiety-like behavior [20, 74, 75], which may contribute to the findings in this study. Future studies are warranted to explore potential sex differences in the role of mPFC^CRF1+^ population.

While preclinical studies suggest that the CRF-CRF1 system is critical in AUD-related behavior, it has not yet been successful in a clinical setting [76, 77]. Therefore, it is imperative to find novel targets that may be translated in the clinic. Here, we rationalized the mPFC^CRF1+^ neurons are responsive to the brain stress signal CRF and used transcriptomics to identify other potential molecular targets for therapeutic intervention. Our findings suggest that CSF1, which has been a target of interest for cancers, Alzheimer’s disease, and other disorders [78, 79], may be a novel target to alleviate negative affect during withdrawal from chronic alcohol exposure contributing to relapse-like behavior.

## Supplementary information

Supplementary Material
