# Supplementary material for: Ethanol withdrawal-induced adaptations in prefrontal corticotropin releasing factor receptor 1-expressing neurons regulate anxiety and conditioned rewarding effects of ethanol
Source: Mol Psychiatry. 2022 Jun 6;27(8):3441–51. doi: 10.1038/s41380-022-01642-3 (PMC9708587; doi:10.1038/s41380-022-01642-3)
Supplement: Supplementary file 1 — Supplementary Material [file 41380_2022_1642_MOESM1_ESM.docx]

**Supplementary Materials**

**Supplementary Methods**

***Animals***

Adult (minimum age of 10 weeks) male CRF1:GFP and CRF1:Cre mice were breed in-house[1-5]. Adult, female CRF1-IRES-Cre mice were used for behavioral testing of the effects of CSF1 overexpression in mPFC^CRF1+^ neurons shown in **Fig. 5**. Mice were group-housed in a temperature and humidity-controlled vivarium on a 12-hour reversed light/dark cycle (lights turn off at 8AM) with food and water available *ad libitum*. All procedures involving the use of experimental animals in this study were approved by The Scripps Research Institute Institutional Animal Care and Use Committee and were consistent with the National Institutes of Health Guide for the Care and Use of Laboratory Animals.

***Chronic intermittent ethanol exposure***

To induce ethanol dependence, male mice were exposed to 5 to 6 consecutive weeks of chronic intermittent ethanol inhalation (CIE) as previously described [3]. Briefly, mice in the dependent group were intraperitoneally injected with 1.75 g/kg alcohol + 68.1 mg/kg pyrazole (alcohol dehydrogenase inhibitor) and placed in vapor chambers for 4 days (16 hours vapor on, 8 hours off). On the third or fourth day of vapor exposure tail blood was collected to determine blood ethanol levels (BELs). The average blood ethanol level achieved was 183.5mg/dl during the weeks of ethanol vapor exposure. Naïve mice were injected with 68.1 mg/kg pyrazole in saline and were placed in CIE chamber but only received air inhalation for the same time. Experiments were conducted from naïve mice, dependent mice directly from the vapor chambers, and withdrawn mice were 5-8 days into forced abstinence.

***In situ hybridization***

Mice were anesthetized with isoflurane and transcardially perfused with cold phosphate-buffered saline (PBS) and 4% paraformaldehyde (PFA). Following dissection, brains were immersion fixed in PFA for 24 hours at 4^o^C and cryoprotected in 30% sucrose in PBS for 24 hours (or until brains sank in the cryoprotectant) at 4^o^ C. Brains were then flash frozen in pre-chilled isopentane on dry ice and stored at -80^o^ C until they were sliced into 20 µm thick sections on a cryostat, mounted on SuperFrost Plus slides (Fisher Scientific, 1255015), and stored at -80^o^C until use. In situ hybridization was performed using RNAscope fluorescent multiplex kit (ACD, 320850) as previously described[1]. A target retrieval pretreatment protocol as outlined in the RNAscope manual (ACD, doc.no. 320535) was followed. Briefly, slides were submerged in target retrieval buffer (ACD, 322000) at 95-98^o^C for 10 mins, immediately rinsed in distilled water, dehydrated in 100% ethanol (storage at -80^o^C if required), and followed by protease IV application to slides for 20 mins at 40^o^C. Next, the RNAScope Fluorescent Multiplex Reagent Kit User Manual (ACD, doc.no. 320293) was followed, and slides were mounted with Vectashield+DAPI (Fisher Scientific, NC9029229). The probes used from ACD Biotechne were as follows: negative control (320751), Crhr1 (418011), Gad2 (439371), and Slc17a7 (416631).

Image acquisition was performed using a Zeiss LSM 780 laser scanning confocal microscope (Jena, Germany) of prelimbic mPFC (40X oil immersion, 1024x1024pixel, 5-μm z-stacks). All microscope settings were kept the same within experiments during image acquisition. Quantification was performed using Fiji[6] by identifying nuclei based on DAPI staining in the mPFC. Nuclei were considered positive for probe if corresponding fluorescent signal was present after background (negative control) subtraction. The percent of positive nuclei for labeled cells was calculated, and in instances of multiple treatment groups normalized to the control/naïve group to show relative values. *Crhr1* imaging was restricted to the medial prefrontal cortex prelimbic layer 2/3 subregion to coincide with electrophysiological recordings. Multiple images were acquired from multiple slices per mouse, and the sample sizes are represented as images per animal. Note, negligible *Crhr1* fluorescence was observed in the mouse septum (see **Supple. Fig. 1**; modified from Wolfe *et al*.[1]), a region of low CRF1 expression [7], confirming the validity of the *Crhr1* signal. Analysis as performed on raw images, and brightness, contrast, and pixel dilation are the same for all representative images shown in figures.

***Immunohistochemistry***

Brain sections were prepared (as described above for *in situ* hybridization) and used for verification of injections sites. For histology, antigen retrieval was performed with Tris-EDTA buffer (10mM Tris Base, 1mM EDTA, 0.05% Tween 20, pH 9.0) at 95-100°C for 10 mins and cooled at room temperature for 20 mins. Then, slides were washed 3 times for 10 mins in PBST (PBS, 0.2% triton x100) and blocked [10% normal donkey serum (NDS), 0.5% triton x100 in PBS] for 2 hrs at room temperature. Slides were then incubated overnight at 4^o^ C with primary antibodies for rabbit anti-CSF1 (1:500; ab233387; Abcam), rabbit anti-Cre (1:500; 69050; Novagen), or chicken anti-GFP (1:2000; ab13970; Abcam) in antibody diluent (2% NDS, 0.1% triton x100 in PBS), washed 3 times for 10 mins in PBST (PBS, 0.2% triton x100), incubated for 2 hrs at room temperature in Alexa Fluor 488 donkey anti-rabbit (Jackson ImmunoResearch, 711-545-152), Alexa Fluor 555 donkey anti-rabbit (1:2000, A32794, Invitrogen), or Alexa Fluor 488 donkey anti-chicken (1:2000, 703-545-155, Jackson ImmunoResearch) in antibody diluent, and washed in PBST again before mounting and cover slipping in Vectashield+DAPI (Fisher Scientific, NC9029229). The prelimbic mPFC was imaged using a Vectra Polaris Imaging system (CLS143455) (40X, 1366x600pixel) or Keyence BZX700 (20X). All microscope settings were kept the same within experiments during image acquisition. Analysis and statistics performed as described above for *in situ* hybridization. Brightness and contrast are the same for all representative images shown in figures.

***Slice electrophysiology***

*Slice preparation[8, 9]*: Mice were anesthetized with 3-5% isoflurane, decapitated, and the brains were quickly removed and placed in ice-cold oxygenated (95% O2 and 5% CO2) high-sucrose cutting solution containing (in mM): 206 sucrose, 2.5 KCl, 2.5 CaCl2, 7 MgCl2, 1.2 NaH2PO4, 26 NaHCO3, 5 glucose, 5 HEPES; pH 7.3. Coronal slices (300 μM) containing the mPFC and BLA were sliced using a 1200S vibratome (Leica Microsystems, Buffalo Grove, IL) and incubated in artificial cerebrospinal fluid (ACSF) containing (in mM): 130 NaCl, 3.5 KCl, 1.25 NaH2PO4, 1.5 MgSO4, 2 CaCl2, 24 NaHCO3, 10 glucose; pH 7.3 at 32°C for 30 mins and then at room temperature for at least 30 mins before use.

*Whole-cell electrophysiological recordings*: Neurons in the prelimbic subdivision of the mPFC were visualized with infrared differential interference contrast (IR-DIC) optics, and fluorescently labeled neurons were identified using Prior LED optics (Prior Scientific, Rockland, MA). Whole-cell voltage-clamp and current-clamp recordings were obtained using Multiclamp 700B amplifier, Digidata 1440A and pClamp 10 software (Molecular Devices, Sunnyvale, CA). Glass pipettes were pulled (PC-10, Narishige) to a resistance of 3-6 MΩ and filled with an internal solution containing (in mM): 135 K-gluconate, 5 EGTA, 2 MgCl2, 10 HEPES, 2 Mg-ATP, 0.2 Na-GTP; 290-300 mOsms; pH 7.2-7.3. AMPA mediated spontaneous excitatory postsynaptic currents (sEPSCs) were recorded using a holding potential of -70mV for experiments in **Fig. 1** and **Fig.** **3**. AMPA mediated miniature excitatory postsynaptic current (mEPSCs) were recorded with a holding potential of -70mV in the presence of 30µM bicuculline (BIC; Tocris) and 0.5µM tetrodotoxin (TTX; Sigma Aldrich) for experiments in **Fig. 5**. Neuronal properties and excitability were assessed using 1 second current injections ranging from -120pA to 200pA in 20pA increments and 1 second current injections ranging from 0pA to 300pA in 10pA increments from a holding potential of -70mV in current-clamp recordings for experiments in ACSF without synaptic blockers in Fig. 1 and 3. Series resistance was not compensated, and cells with a series resistance > 20 MΩ or with a > 20% change during the recording were excluded.

*Ex vivo optogenetic experiments*: Slices containing the BLA were first visualized under Lumen 300 LED optics (Prior Scientific) to confirm ChR2-mCherry injection sites in all BLA slices prior to electrophysiological recordings. Mice with missed injection sites were excluded from experiments. To confirm functional activity of ChR2, ChR2-photocurrents were recorded from mCherry expressing BLA neurons using wide-field illumination (1s or 3ms; 10, 20, 40Hz trains, 470nM, ~10mW) through a 40X water immersion objective in voltage-clamp mode from a holding potential of -70mV as seen in Fig. 4B. Mono-synaptic connectivity between the BLA and mPFC^CRF1+^ neurons was measured using wide-field illumination (3ms, 470nM, ~10mW) of ChR2-mCherry BLA terminals in the mPFC through a 40X water immersion in ACSF containing 30µM BIC, 0.5µM TTX, and 100µM 4-aminopyridine (4-AP) as seen in **Fig. 3** and **Supple. Fig. 5.** AMPA and NMDA currents were recorded using an internal solution containing (in mM): 145 Cs-methanesulfonate, 10 HEPES, 5 EGTA, 2 MgCl_2_, 2 Mg-ATP, 0.2 Na-GTP, 10 phosphocreatine, 5 QX-314; pH7.2-7.3 and from a holding potential of -80mV and +40mV, respectively. Data were analyzed using Mini Analysis (Synaptosoft Inc., Fort Lee, NJ) and Clampfit 10.7 (Molecular Devices), and all events were visually confirmed. Peak NMDA current was calculated at 50 ms following optogenetic stimulation. To obtain average baseline s/mEPSC, characteristics, events were binned into either 1 or 3 min bins depending on the experiment with a minimal inclusion of 100 events. Measurements for the effects of acute CRF (Tocris) application were made after 9 mins of CRF application and normalized to a pre-CRF application baseline.

***Site-specific viral injection surgery***

Mice were anesthetized with 5% isoflurane, secured in a stereotaxic frame (David Kopf Instruments, Tujunga, CA), and maintained on 1-2% isoflurane during surgery. Viruses were bilaterally injected in the brain at 100nL/min for a total volume of 300nL using either 28-gauge injector cannulas (Plastics One Inc., Roanoke, VA) connected to 10µL Hamilton syringes and a microfluidic syringe pump (Harvard Apparatus, Holliston, MA). Specifically, for optogenetic-assisted circuit dissection AAV2-hSyn-ChR2-mCherry was injected into the BLA (AP: -1.5, DV: -4.9, and ML: ±3.4 from dura) (UNC Vector Core, Chapel Hill, NC), for mPFC^CRF1+^ ablation AAV2-flex-taCasp3-TEVp (UNC Vector Core), for mPFC^CRF1+^ CSF1 overexpression AAV2-CMV-DIO-mCSF1-2A-mCherry or AAV2-CMV-DIO-mCSF1-2A-GFP (Vector Biolabs, Malvern PA), or control AAV2-hSyn-DIO-mCherry or AAV2-hSyn-DIO-GFP (Addgene) was injected into the mPFC (AP: +1.9, DV: -2.4, and ML: ±0.5 from dura). After injection, cannulas were kept in place for an additional 10mins before slowly removing and the skin being sutured. Mice remained group-housed and underwent a recovery period of 4 weeks before experimental testing. Following experimental testing, all brains were collected and processed for immunohistochemical analysis to confirm injection. Mice with missed injection sites were excluded from the dataset.

***Behavioral testing***

All behavioral testing (specified below) was conducted early in the dark phase. CRF1:Cre male mice were used for behavioral testing following ablation of mPFC^CRF1+^ neurons, and CRF1:Cre female mice were used for behavioral testing following overexpression of CSF1 in mPFC^CRF1+^ neurons.

*Novel object recognition:* Mice were individually habituated to an open field for 5 mins, removed and then re-exposed, now with two identical objects placed in the field, for 5 mins. Mice were briefly removed to clean the arena and tested a second time with the same two objects. Finally, mice were re-exposed to the arena containing one familiar and one novel object for 5 mins. The objects were of comparable height and volume but different in shape and appearance, and location of novel and familiar objects were alternated among the mice. The time spent interacting with each object was manually scored from video recordings.

*Novelty-induced suppression of feeding:* Following 24 hours of food deprivation, mice were placed in a novel, open arena (50 cm X 50 cm) (center lux of 400) with a piece of familiar food in the center, and the latency to feed was measured. Immediately after mice began to feed in the open arena, they were moved to their home cage (red light) and the same piece of food was placed in the center, and the latency to feed was again measured.

*Three-chamber social interaction and novelty test:* The social interaction apparatus consists of a three-chambered Plexiglas box with each chamber measuring length 20 cm x width 40.5 cm x height 22 cm and separated by clear walls with a small opening allowing access into each chamber. First, mice were habituated to the entire apparatus for 5 mins. Then the middle chamber was kept empty, but small, round wire cages (Galaxy Cup, Spectrum Diversified Designs, Inc., Streetsboro, OH) were placed in each of the two outer chambers. To assess sociability, mice were returned to the middle chamber with a stranger C57BL6J mouse of the same sex being tested (habituated to the wire cage) in one of the wire cages in an outer compartment and the other identical, but empty, wire cage in the opposite compartment. Time spent in the chamber with the stranger mouse and time spent in the chamber with the novel object were recorded for 5 min. For the social novelty preference test, mice were returned to the middle chamber, this time with the original mouse (familiar mouse) in its chamber and a new unfamiliar mouse (novel mouse) in the previously empty wire cage. Time spent in each chamber was recorded for 5 min.

*Ethanol place conditioning:* The conditioned place apparatus is a Plexiglas black matte box (44 x 22 x 30 cm) divided by central partitions into two chambers of equal size (22 x 22 x 30 cm), each with distinctive tactile stimuli (smooth and textured). Mice underwent three (in red light) phases: pre-conditioning, conditioning and test. During the pre-conditioning phase, mice were allowed to freely move in the entire place conditioning apparatus for a single 30 min session. Mice that showed unbiased exploration of the two sides of the apparatus during pre-conditioning (between 45 and 55% time spend on each side) were randomly assigned a side in which to receive ethanol. In the 12-day conditioning phase, mice received i.p. injections of 2g/kg ethanol (20% w/v) or saline and were immediately confined to one compartment of the place conditioning apparatus for 30 mins. Treatments and compartment sides were alternated each day, such that each mouse experienced 6 pairing of ethanol with one side of the apparatus. On the test day, mice were given 30 min access to the entire place conditioning apparatus in a drug-free state. The time spent in each of the two compartments was measured during the pre-conditioning and test phases by analysis of the video recording using Noldus Ethovision software.

***Fluorescence activated cell sorting and RNA sequencing***

*Dissociation of mPFC neurons and isolation of mPFC^CRF1+^ neurons using fluorescence activated cell sorting:* Male naïve and withdrawn CRF1:GFP mice were used for transcriptomic analysis. The mPFC from each mouse was quickly microdissected in ice cold neuronal media (containing Hibernate A media (Gibco A1247501), 2% B27 supplement (Gibco 12587010) and 0.25% glutamax (Gibco 35050061)) using a mouse brain matrix, and tissue was cut into small pieces with a scalpel blade. Tissue was dissociated as previously described[11]. Briefly, cells were enzymatically digested using papain-containing L-cysteine (PAP2 10 U ml^-1^, Worthington Biochemical) for 15 min at 32°C. Every 5 mins during digestion, tissue was mechanically triturated. The cell suspension was then transferred onto an Opti-prep density gradient prepared for neurons and detailed in[12], centrifuged, cell pellets were washed and resuspended in neuronal media. Resuspended neurons were strained through a 35µM mesh filter to obtain single-cell suspensions for sorting and kept on ice. Prior to sorting, neuronal suspensions were stained with 1µM DAPI (Sigma Aldrich) and 1µM DRAQ5 (Cell Signaling Technology) for a minimum of 10mins. Appropriate gates for FACSs were set based on GFP, DAPI, and DRAQ5 intensity to sort live mPFC^CRF1+^ neurons using MoFlo Astrios (Beckman Coulter). Cells were sorted directly into Trizol LS (Invitrogen).

*RNA isolation and sequencing:* Total RNA was isolated from FACS-sorted cells using Direct-zol RNA MiniPrep Kit (Zymo Research) according to the manufacturer’s protocol. Linearized acrylamide (1µg) was added to each sample before processing. Agilent 2100 Bioanalyzer was used to confirm quality and quantity of RNA for each sample. Each sample represents the mPFC from a single mouse. 160pg of RNA from each sample taken into the SMARTseq HT library prep kit (Takara).  14 PCR cycles were used to generate cDNA.  PCR-amplified cDNA was purified by AMPure XP beads (Beckman).  The cDNA was quantified by Qubit and QC'd by bioanalyzer HS.  cDNA was converted into sequencing libraries following SMARTseq HT modified Illumina Nextera XT DNA protocol, 100pg cDNA taken into the kit.  12 PCR cycles were used to generate tagmented cDNA libraries.  The libraries were pooled and purified by AMPure XP beads.  The tagmented cDNA pooled library was quantified by Qubit and QC'd by bioanalyzer High Sensitivity chip.  Each sample was sequenced to ~20M  1 x 75 reads. The reads were trimmed for the adapter sequences using cutadapt 1.18[13] with Python 3.6.3. The trimmed reads were mapped to the reference genome (Mouse ENSEMBL GRCm38 revision^91^) using the STAR aligner 2.5.2a[14]. Gene abundance was estimated with python 2.7.11, and HTSeq 0.11.0[15]. The differential gene expression analyses was performed using R 3.5.1, and DESeq2 1.20.0[16]. Distribution of mapped reads over genome features was obtained for each sample using RSeQC 2.6.4. Read quality was assessed with fastqc. Any adapter sequence was trimmed using cutadapt. Genes were retained as significantly differentially expressed when meeting following thresholds of adjusted *p* value ≤ 0.05 and log_2_ fold change > 0.6. Advaita bioinformatics was used for gene ontology, pathway and network analysis. Raw and processed data will be available on Gene Expression Omnibus (GEO).

***Data analysis***

In situ hybridization, electrophysiological and behavioral data were graphed and statistical analyses were performed using Graphpad Prism (San Diego, CA) and OriginPro 2017 (OriginLab Corp., Northampton, MA). Data are presented as mean ± standard error (SEM) with individual data points overlayed, and *N and n* represents sample number of mice and cells, respectively. Sample sizes were choosen based on previously conducted experiments. Grubb’s outlier test was used to identify outliers, which were excluded from datasets. All statistical tests, stated in the figure legend for each experiment, met the appropriate assumption regarding normal distribution and homoscedasticity of data, were two-tailed, and p values were adjusted for multiple comparisons as appropriate. A *p* value of < 0.05 was considered the cutoff for significance.

**Supplementary Tables**

**Supplementary Table 1. Sample size for experiments in the manuscript.**

**Supplementary Table 2. Electrophysiological properties of** **mPFC^CRF1-^ and mPFC^CRF1+^ neurons from naïve, ethanol dependent, and withdrawn mice.** Statistical significance **p* < 0.05, ***p* < 0.01, and ****p* < 0.001 from one-way ANOVA post hoc comparison to naïve.

|  |  | **Rheobase (pA)** | **Input Resistance (MOhm)** | **Action Potential Amplitude (mV)** | **Capacitance (pF)** | **Resting Membrane Potential (mV)** |
| --- | --- | --- | --- | --- | --- | --- |
| **mPFC^CRF1-^** | **Naïve (*n* = 35)** | 72.5 ± 6.7 | 296 ± 23.0 | 85.7 ± 1.1 | 174.4 ± 8.2 | -69.1 ± 0.2 |
|  | **Dependent (*n* = 25)** | 76.0 ± 8.3 | 287.6 ± 22.5 | ******90.8 ± 1.2 | 165.6 ± 4.4 | -68.9 ± 0.14 |
|  | **Withdrawn (*n* = 24)** | 63.6 ± 7.7 | 286.6 ± 36.6 | 86.4 ± 1.1 | 182.2 ± 9.1 | *****-68.3 ± 0.3 |
| **mPFC^CRF1+^** | **Naïve (*n* = 40)** | 68.7 ± 7.3 | 295.6 ± 15.8 | 89.4 ± 0.6 | 176.1 ± 7.7 | -69.6 ± 0.2 |
|  | **Dependent (*n* = 29)** | 64.5 ± 6.1 | 290.0 ± 15.8 | 90.2 ± 0.6 | 184.0 ± 7.0 | -69.5 ± 0.1 |
|  | **Withdrawn (*n* = 31)** | 71.0 ± 7.7 | 320.9 ± 19.1 | *******86.1 ± 0.7 | 156.5 ± 6.7 | -69.2 ± 0.1 |

**Supplementary Table 3. Electrophysiological properties of mPFC^CRF1-^ and mPFC^CRF1+^ neurons during baseline and following CRF (200nM) application.** Statistical significance **p* < 0.05*, **p <* 0.01*, ***p* < 0.001 from two-way ANOVA post hoc comparison to baseline.

|  |  | **Rheobase (pA)** | **Input Resistance (MOhm)** | **Action Potential Amplitude (mV)** | **Capacitance (pF)** | **Resting Membrane Potential (mV)** |
| --- | --- | --- | --- | --- | --- | --- |
| **mPFC^CRF1-^** | **Baseline (*n* = 9)** | 49.6 ± 5.7 | 306.6 ± 23.0 | 90.9 ± 1.4 | 168.2 ± 22.6 | -72.6 ± 1.0 |
|  | **CRF (*n* = 9)** | *86.5 ± 12.9 | **234.2 ± 21.4 | 92.9 ± 2.0 | 158.1 ± 11.1 | -71.1 ± 0.9 |
| **mPFC^CRF1+^** | **Baseline (*n* = 11)** | 65.5 ± 12.0 | 292.7 ± 35.3 | 94.3 ± 1.1 | 176.0 ± 24.5 | -71.9 ± 0.8 |
|  | **CRF (*n* = 11)** | **121.9 ± 24.4 | ***204.4 ± 28.8 | 93.9 ± 0.9 | 187.4 ± 14.7 | -71.3 ± 0.6 |

**Supplementary Table 4. List of all significantly impacted pathways.**

| **Pathway Name** | **p-value** |
| --- | --- |
| Phagosome | 1.01E-09 |
| Graft-versus-host disease | 4.25E-06 |
| Type I diabetes mellitus | 6.53E-06 |
| Viral myocarditis | 7.06E-06 |
| Human papillomavirus infection | 7.58E-06 |
| PI3K-Akt signaling pathway | 7.85E-06 |
| Human T-cell leukemia virus 1 infection | 8.11E-06 |
| Antigen processing and presentation | 9.09E-06 |
| Allograft rejection | 1.3E-05 |
| Autoimmune thyroid disease | 1.62E-05 |
| Protein digestion and absorption | 2.05E-05 |
| Cell adhesion molecules (CAMs) | 2.33E-05 |
| Focal adhesion | 2.42E-05 |
| Tuberculosis | 4.52E-05 |
| ECM-receptor interaction | 8.86E-05 |
| Metabolic pathways | 0.000117 |
| Fc gamma R-mediated phagocytosis | 0.000119 |
| Proteoglycans in cancer | 0.000126 |
| Leishmaniasis | 0.000402 |
| Pathways in cancer | 0.000503 |
| Adherens junction | 0.000543 |
| TGF-beta signaling pathway | 0.000666 |
| Cellular senescence | 0.000729 |
| Epstein-Barr virus infection | 0.000862 |
| Rheumatoid arthritis | 0.001091 |
| Hypertrophic cardiomyopathy (HCM) | 0.001218 |
| Axon guidance | 0.00152 |
| Asthma | 0.001607 |
| Hematopoietic cell lineage | 0.001644 |
| Insulin resistance | 0.001861 |
| Estrogen signaling pathway | 0.001988 |
| Prostate cancer | 0.002076 |
| Basal cell carcinoma | 0.002103 |
| Inflammatory bowel disease (IBD) | 0.002228 |
| Hedgehog signaling pathway | 0.00224 |
| Ras signaling pathway | 0.002326 |
| Dilated cardiomyopathy (DCM) | 0.002642 |
| Choline metabolism in cancer | 0.002761 |
| Toxoplasmosis | 0.003114 |
| Endocytosis | 0.003213 |
| Human cytomegalovirus infection | 0.003229 |
| p53 signaling pathway | 0.003694 |
| Arrhythmogenic right ventricular cardiomyopathy (ARVC) | 0.003875 |
| Intestinal immune network for IgA production | 0.004607 |
| Viral carcinogenesis | 0.005009 |
| Cushing syndrome | 0.005774 |
| cAMP signaling pathway | 0.006324 |
| Human immunodeficiency virus 1 infection | 0.006967 |
| Breast cancer | 0.008326 |
| Regulation of actin cytoskeleton | 0.009273 |
| Ether lipid metabolism | 0.009307 |
| VEGF signaling pathway | 0.009327 |
| Hepatocellular carcinoma | 0.009812 |
| Sphingolipid metabolism | 0.009861 |
| Transcriptional misregulation in cancer | 0.010304 |
| Parathyroid hormone synthesis, secretion and action | 0.010453 |
| GnRH signaling pathway | 0.010546 |
| Fatty acid degradation | 0.011026 |
| Hippo signaling pathway | 0.011512 |
| Melanogenesis | 0.012078 |
| Glycosaminoglycan biosynthesis - chondroitin sulfate/dermatan sulfate | 0.014402 |
| Gastric cancer | 0.014619 |
| Amphetamine addiction | 0.015191 |
| Amoebiasis | 0.016224 |
| Renin-angiotensin system | 0.016563 |
| Alcoholism | 0.017261 |
| Phospholipase D signaling pathway | 0.017854 |
| Fatty acid metabolism | 0.018001 |
| Leukocyte transendothelial migration | 0.018065 |
| Th17 cell differentiation | 0.018877 |
| Rap1 signaling pathway | 0.01919 |
| Aldosterone synthesis and secretion | 0.019475 |
| Hepatitis B | 0.020345 |
| Th1 and Th2 cell differentiation | 0.020363 |
| alpha-Linolenic acid metabolism | 0.022064 |
| TNF signaling pathway | 0.022275 |
| Wnt signaling pathway | 0.023048 |
| Protein processing in endoplasmic reticulum | 0.023383 |
| Sphingolipid signaling pathway | 0.024722 |
| Drug metabolism - cytochrome P450 | 0.024991 |
| Kaposi sarcoma-associated herpesvirus infection | 0.026519 |
| Relaxin signaling pathway | 0.03003 |
| Pancreatic cancer | 0.03029 |
| Glioma | 0.03474 |
| MAPK signaling pathway | 0.035675 |
| Signaling pathways regulating pluripotency of stem cells | 0.038306 |
| Ubiquitin mediated proteolysis | 0.040054 |
| Herpes simplex virus 1 infection | 0.040915 |
| Ferroptosis | 0.040939 |
| Vasopressin-regulated water reabsorption | 0.041754 |
| Nicotinate and nicotinamide metabolism | 0.043379 |
| Bacterial invasion of epithelial cells | 0.044875 |
| Salmonella infection | 0.044993 |
| Arachidonic acid metabolism | 0.04927 |

**Supplementary Table 5. Electrophysiological properties of mPFC^CRF1-^ and mPFC^CRF1+^ neurons from control and CSF1 overexpression mice.** Statistical significance **p* < 0.05 by unpaired t-test compared to control.

|  |  | **Rheobase (pA)** | **Input Resistance (MOhm)** | **Action Potential Amplitude (mV)** | **Capacitance (pF)** | **Resting Membrane Potential (mV)** |
| --- | --- | --- | --- | --- | --- | --- |
| **mPFC^CRF1-^** | **Control (*n* = 13)** | 73.3 ± 10.5 | 258 ± 25.0 | 73.3 ± 4.7 | 119.9 ± 14.7 | -72.5 ± 0.5 |
|  | **CSF1 (*n* = 9)** | 80.23 ± 13.2 | 249.9 ± 41.4 | 77.7 ± 2.5 | 140.9 ± 18.5 | -72.6 ± 0.9 |
| **mPFC^CRF1+^** | **Control (*n* = 39)** | 71.1 ± 4.8 | 226.6 ± 18.2 | 81.2 ± 2.5 | 148.2 ± 7.9 | -71.2 ± 0.4 |
|  | **CSF1 (*n* = 29)** | 87.0 ± 6.7 | 222.6 ± 18.6 | 75.1 ± 3.4 | *121.4 ± 9.9 | -70.7 ± 0.8 |

**Supplementary Figures**


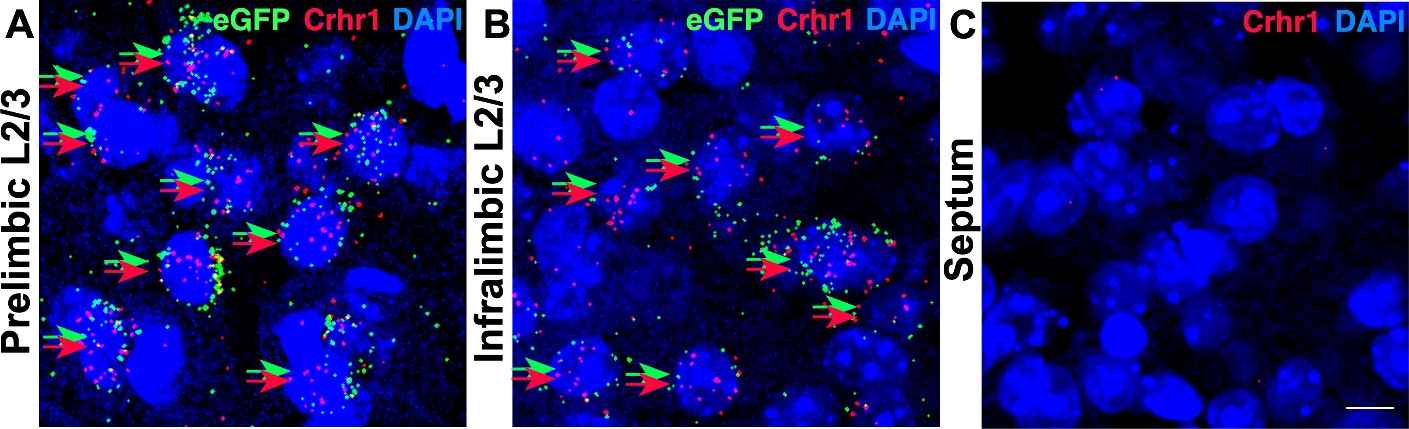


**Supplementary Figure 1. CRF1 labeling in the mPFC in a CRF1:GFP reporter mouse.** Representative image of **A.** Prelimbic L2/3 and **B.** Infralimbic L2/3 in CRF1:GFP mice showing eGFP mRNA co-localizes with CRF1 mRNA (Crhr1) mRNA confirming specificity of GFP in Crhr1+ nuclei. **C.** Crhr1 probe specificity was validated in the septum of wild type mice, a region of low Crhr1/GFP expression. Negligible expression was observed in the septum. (GFP=green, Crhr1=red, and DAPI=Blue; scale=10μm).

**
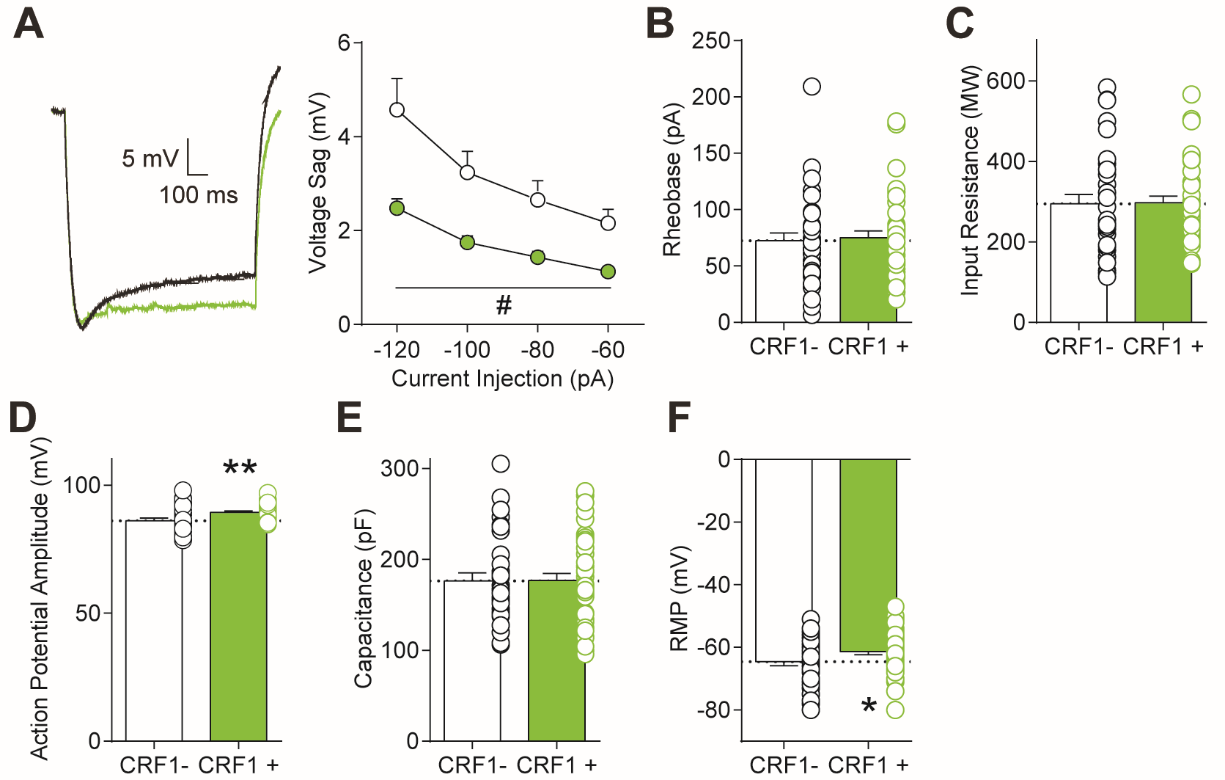
**

**Supplementary Figure 2. A.** Representative voltage traces elicited by -120 pA current injection depicting voltage sag, and average magnitude of voltage sag, calculated as the difference between the peak and plateau voltages, with increasing current injections. There were significant effects of voltage F(3, 180) = 106.1, *p* < 0.0001; group F(1,60) = 14.18, *p* = 0.0004; and interaction F(3,180) = 8.72, *p* < 0.0001 by two-way ANOVA, and post hoc significance ^#^*p* < 0.05 is represented in panel. **B-F.** Average rheobase, input resistance, initial action potential amplitude, capacitance, and resting membrane potential in mPFC^CRF1-^ and mPFC^CRF1+^ neurons. *n* = 31-39 cells from 9-10 male mice; **p* < 0.05, ***p* < 0.01 by t-test.

**
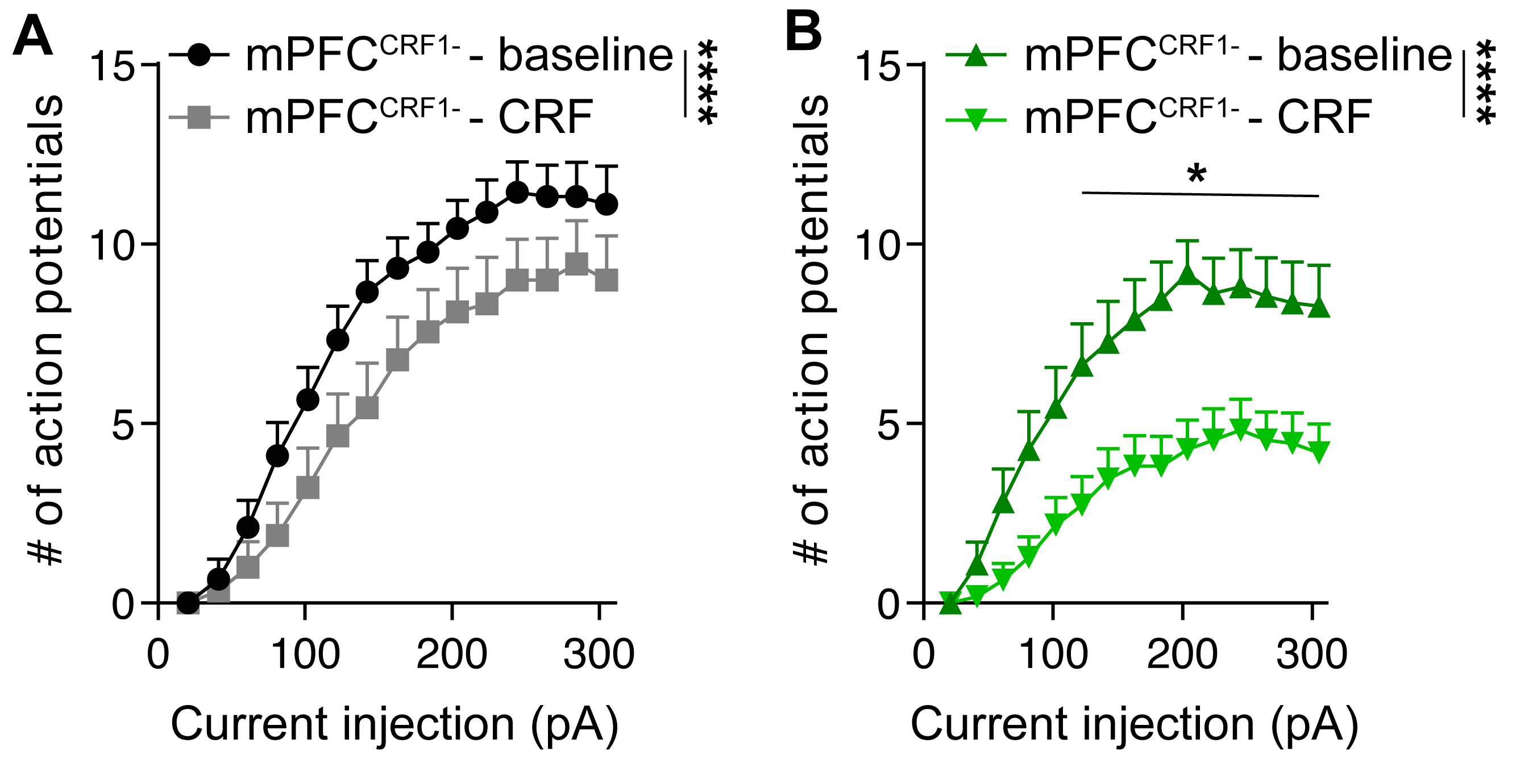
**

**Supplementary Figure 3. CRF (200nM) effects on mPFC prelimbic layer 2/3 neuronal excitability. A.** CRF effects on mPFC^CRF1-^ neuron excitability. **B.** CRF effects on mPFC^CRF1+^ neuron excitability. *n* = 9-11 cells from *N* = 3 male mice; *****p* < 0.0001 main effect by two-way ANOVA, **p* < 0.05 post hoc multiple comparisons.


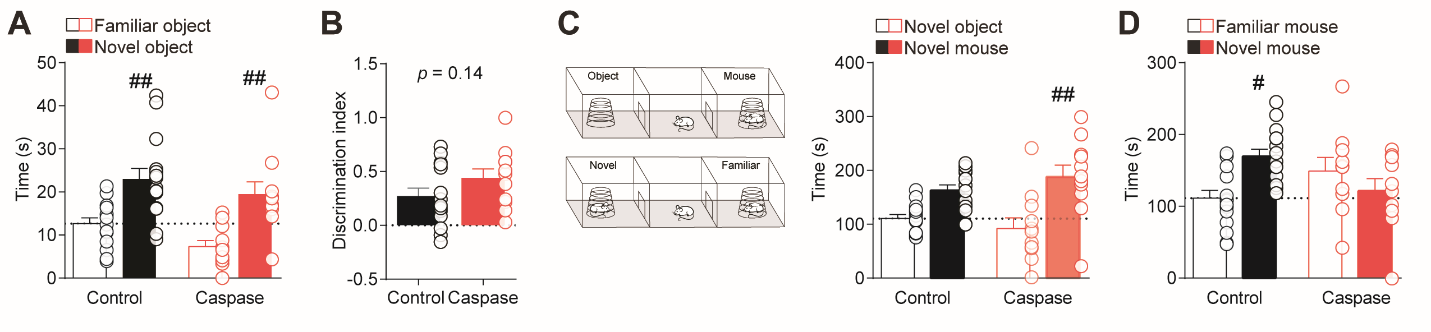
**Supplementary Figure 4. mPFC^CRF1+^ ablation does not alter novel object recognition and abolishes social novelty preference.** **A.** Average interaction time with a familiar (open bars) and novel (filled bars) object in the novel object recognition test in control (black) and mPFC^CRF1+^ ablated (red) mice with significant main effect of object F(1,24) = 31.79, *p* < 0.0001 by two-way ANOVA, and post hoc significance ^##^*p* < 0.01 is represented in panel. **B.** Average discrimination index calculated as the difference in interaction time between the novel and familiar object divided by the total object interaction time for control (black) and mPFC^CRF1+^ ablated (red) mice. **C.** Average time interacting with a novel object (open bars) and novel mouse (filled bars) in the three-chamber social interaction test in control (black) and mPFC^CRF1+^ ablated (red) mice with a significant main effect of object/mouse F(1,24) = 13.33, *p* = 0.001 by two-way ANOVA, and post hoc significance ^##^*p* < 0.01 represented in panel. **D.** Average time interacting with a familiar mouse (open bars) and novel mouse (filled bars) in the three-chamber social novelty test in control (black) and mPFC^CRF1+^ ablated (red) mice with a significant interaction effect F(1,23) = 5.03, *p* = 0.03 by two-way ANOVA, and post hoc significance ^#^*p* < 0.05 represented in panel. *N* = 10-15 male mice.

**
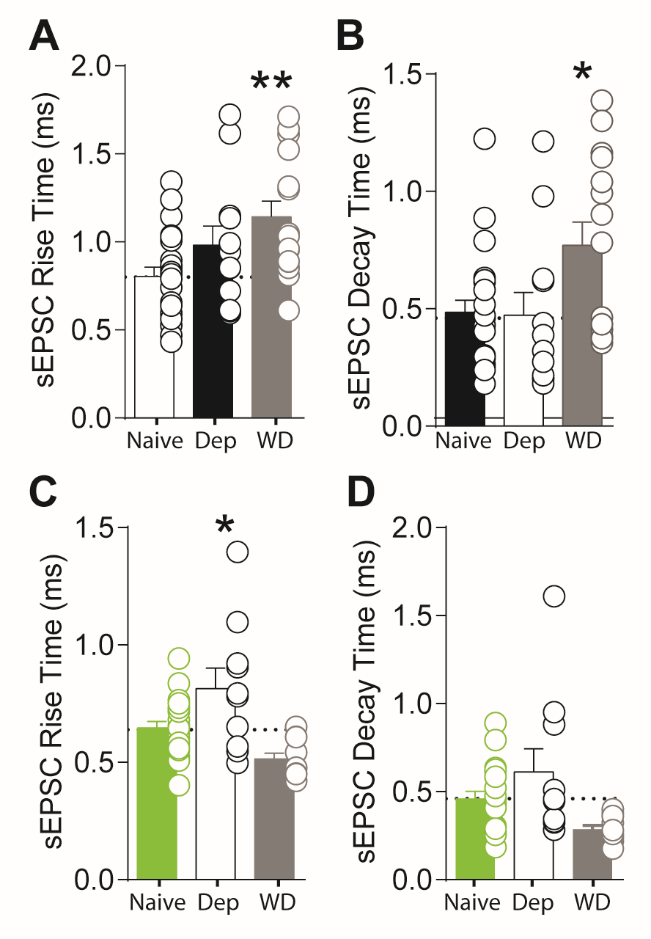
**

**Supplementary Figure 5.** **A, B.** Average sEPSC rise time and decay time kinetics in mPFC^CRF1-^ neurons from naïve (white), dependent (black), and withdrawn (grey) mice. *n* = 12-22 cells from 6-10 mice; **p* < 0.05, ***p* < 0.01 by one-way ANOVA and post hoc multiple comparisons compared to naive. **C, D.** Average sEPSC rise time, and decay time kinetics in mPFC^CRF1+^ neurons from naïve (green), dependent (white), and withdrawn (grey) mice. *n* = 10-21 cells from *N* = 6-10 male mice; **p* < 0.05, ***p* < 0.01 by one-way ANOVA and post hoc multiple comparisons compared to naive.


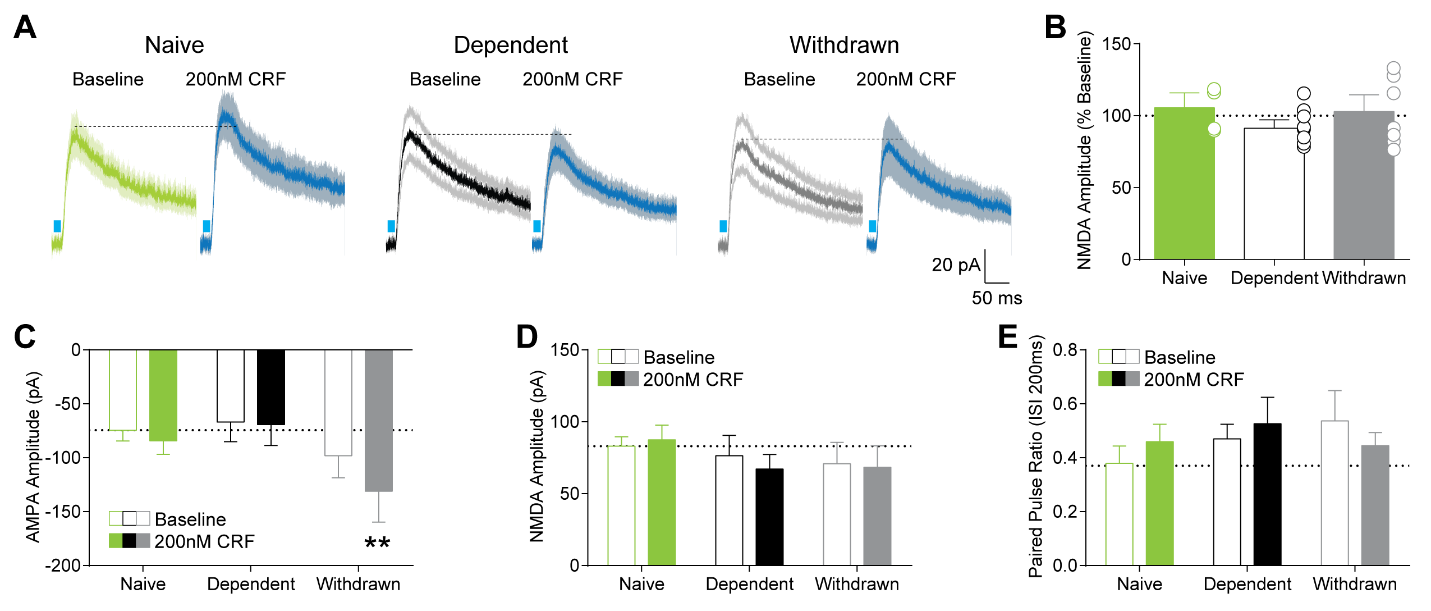


**Supplementary Figure 6. CRF effects on BLA-mediated NMDA *currents*, AMPA currents, and the paired pulse ratio in mPFC^CRF1+^ neurons. A.** BLA-mediated NMDA currents elicited by blue light pulses during baseline (open) and following 200nM CRF (solid) application from a holding potential of -80mV in mPFC^CRF1+^ neurons from naïve, dependent, and withdrawn mice with a significant F(1,17) = 6.92, *p* = 0.01 effect of CRF by one-way ANOVA. **B.** Average baseline normalized NMDA amplitude elicited by optical stimulation of BLA terminals. **C-E.** BLA-mediated AMPA current, NMDA current, and the paired pulse ratio, respectively, during baseline (open) and following 200nM CRF (solid) application in naïve, dependent, and withdrawn mice. A significant F(1,17) = 6.92; *p* = 0.01 effect of CRF on BLA-mediated AMPA currents, and post hoc significance **p* < 0.05 and ***p* < 0.01 is represented in figure.


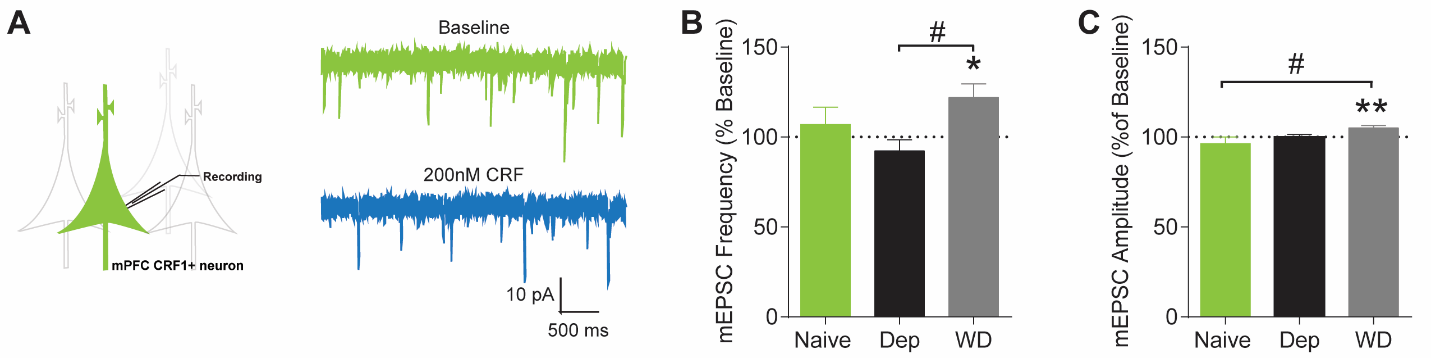


**Supplementary Figure 7. CRF potentiates glutamate transmission selectively in mPFC^CRF1+^ neurons from withdrawn mice. A.** Representative miniature excitatory post-synaptic current traces during baseline (green) and following CRF application (blue) in mPFC^CRf1+^ neurons from naïve mice. **B.** Average mEPSC frequency in mPFC^CRF1+^ neurons from naïve, dependent, and withdrawn mice. **C.** Average mEPSC amplitude in mPFC^CRF1+^ neurons from naïve, dependent, and withdrawn mice. *n* = 4-7 cells from *N* = 3-5 male mice; **p* < 0.05 and ***p* < 0.01 by one sample t-test; ^#^*p* < 0.05 by one-way ANOVA.

**
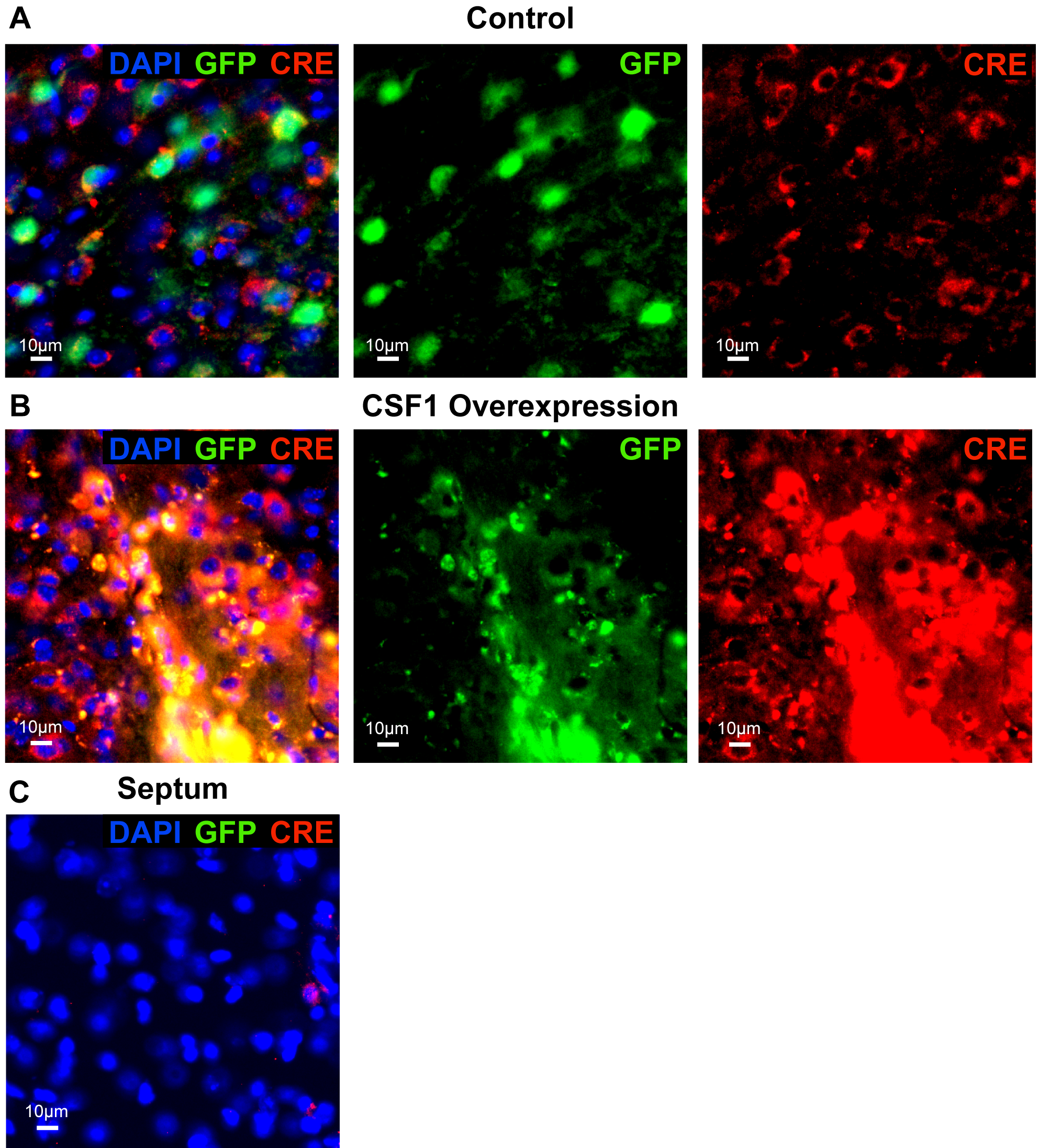
Supplementary Figure 8. Co-expression of GFP selectively in Cre-expressing neurons in the mPFC prelimbic subregion in CRF1:Cre mice.** Representative image of **A.** control injected CRF1:Cre mice, and **B.** CSF1 overexpression injected CRF1:Cre mice. Images indicate expression of GFP labeled virus in Cre positive neurons (GFP=green, Cre=red, and DAPI=Blue; scale=10μm). **C.** Representative image of the septum of CRF1:Cre mice to validate the specificity of the Cre signal.

**
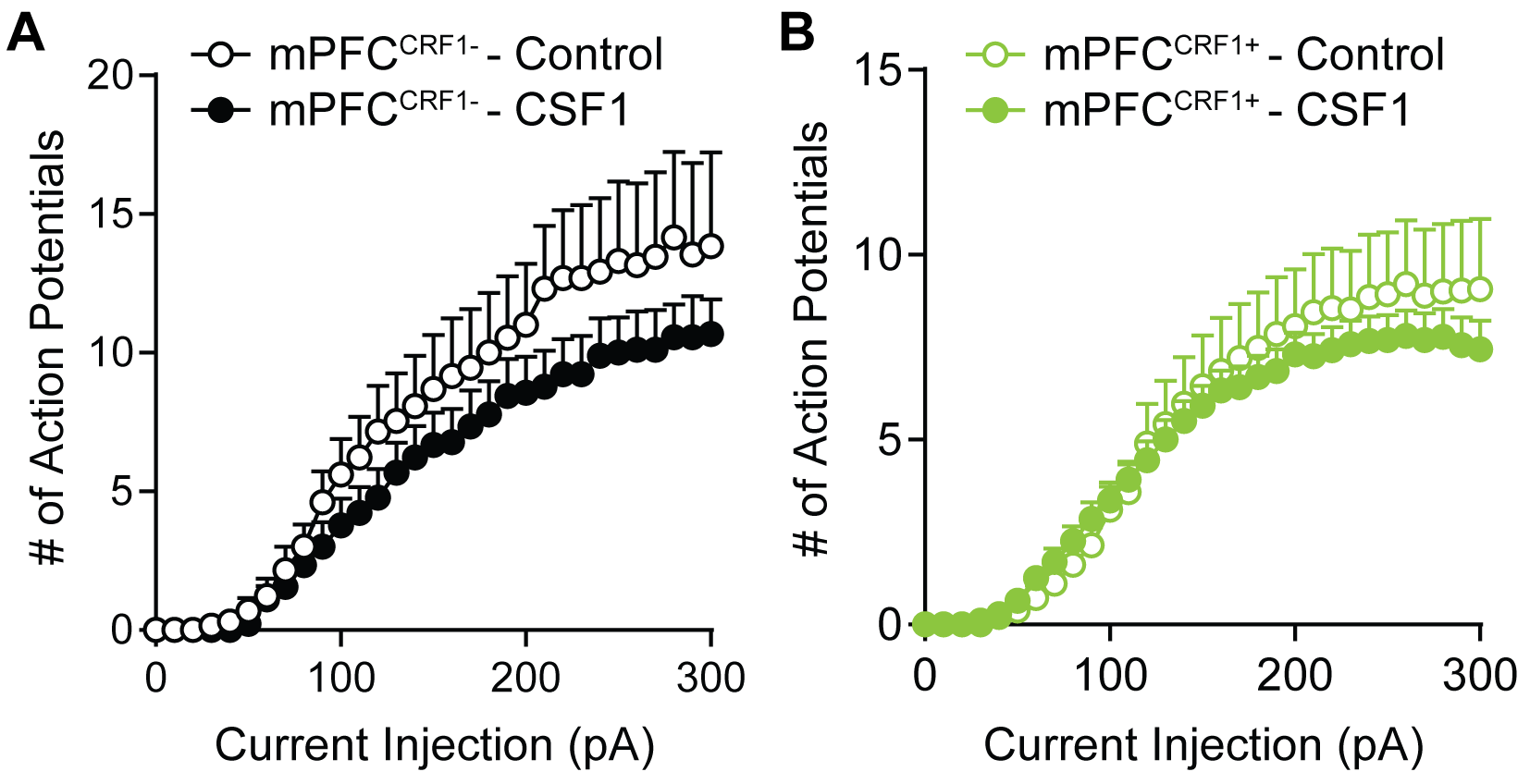
**

**Supplementary Figure 9. CSF1 overexpression in mPFC^CRF1+^ neurons effects on excitability of mPFC^CRF1+^ and mPFC^CRF1-^ neurons. A.** Average number of action potentials elicited by increasing current injections in mPFC^CRF1-^ neurons from naïve (*green diamonds*), dependent (*white diamonds*), and withdrawn (*grey diamonds*) mice with main effects for current injection F(29,2842) = 139.2, *p* < 0.0001; group F(2,98) = 5.60, *p* = 0.005; and interaction effect F(58,2842) = 4.00, *p* < 0.001 by two-way ANOVA from *n* = 29-40 mPFC^CRF1+^ and *n* = 9-13 mPFC^CRF1-^ cells and *N* = 6-10 male mice, and post hoc significance ^#^*p* < 0.05 compared to naive is represented in panel.

**
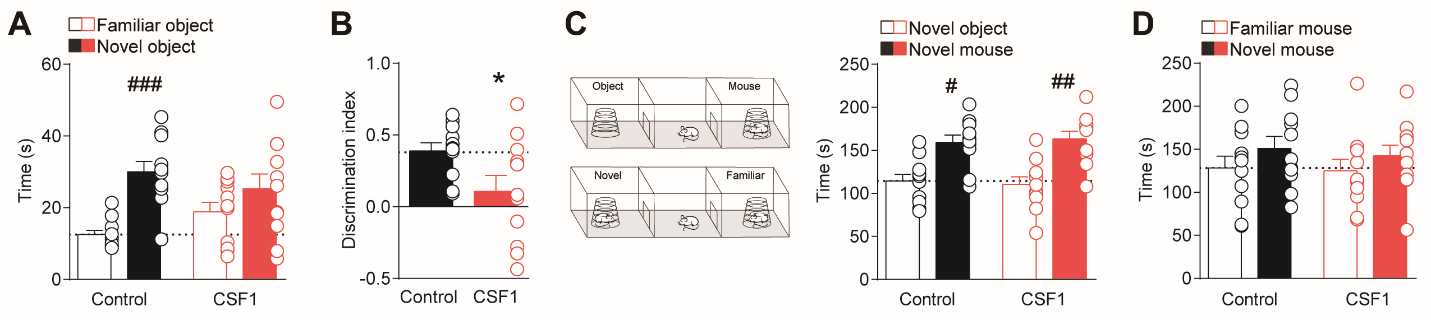
**

**Supplementary Figure 10. CSF1 overexpression in mPFC^CRF1+^ neurons impairs novel object recognition and does not alter sociability. A.** Average interaction time with a familiar (open bars) and novel (filled bars) object in the novel object recognition test in control (black) and CSF1 overexpressing mPFC^CRF1+^ (red) mice with a main effect for object F(1,20) = 19.50, *p* = 0.0003 by two-way ANOVA, and post hoc significance is represented in panel. **B.** Average discrimination index calculated as the difference in interaction time between the novel and familiar object divided by the total object interaction time for control (black) and CSF1 overexpressing mPFC^CRF1+^ (red) mice with a significant effect *p* < 0.05 by unpaired t-test. **C.** Average time interacting with a novel object (open bars) and novel mouse (filled bars) in the three-chamber social interaction test in control (black) and CSF1 overexpressing mPFC^CRF1+^ (red) mice with a significant main effect of object/mouse F(1,20) = 18.27, *p* = 0.0004 by two-way ANOVA, and post hoc significance is represented in panel. **D.** Average time interacting with a familiar mouse (open bars) and novel mouse (filled bars) in the three-chamber social novelty test in control (black) and CSF1 overexpressing mPFC^CRF1+^ (red) mice. *N* = 10-11 female mice.

**Supplementary References**

1. Wolfe, S.A., et al., *Molecular, Morphological, and Functional Characterization of Corticotropin-Releasing Factor Receptor 1-Expressing Neurons in the Central Nucleus of the Amygdala.* eNeuro, 2019. **6**(3).

2. Herman, M.A., et al., *Novel subunit-specific tonic GABA currents and differential effects of ethanol in the central amygdala of CRF receptor-1 reporter mice.* J Neurosci, 2013. **33**(8): p. 3284-98.

3. Herman, M.A., C. Contet, and M. Roberto, *A Functional Switch in Tonic GABA Currents Alters the Output of Central Amygdala Corticotropin Releasing Factor Receptor-1 Neurons Following Chronic Ethanol Exposure.* J Neurosci, 2016. **36**(42): p. 10729-10741.

4. Justice, N.J., et al., *Type 1 corticotropin-releasing factor receptor expression reported in BAC transgenic mice: implications for reconciling ligand-receptor mismatch in the central corticotropin-releasing factor system.* J Comp Neurol, 2008. **511**(4): p. 479-96.

5. Sanford, C.A., et al., *A Central Amygdala CRF Circuit Facilitates Learning about Weak Threats.* Neuron, 2017. **93**(1): p. 164-178.

6. Schindelin, J., et al., *Fiji: an open-source platform for biological-image analysis.* Nat Methods, 2012. **9**(7): p. 676-82.

7. Van Pett, K., et al., *Distribution of mRNAs encoding CRF receptors in brain and pituitary of rat and mouse.* J Comp Neurol, 2000. **428**(2): p. 191-212.

8. Patel, R.R., et al., *IL-1beta expression is increased and regulates GABA transmission following chronic ethanol in mouse central amygdala.* Brain Behav Immun, 2019. **75**: p. 208-219.

9. Warden, A.S., et al., *Microglia Control Escalation of Drinking in Alcohol-Dependent Mice: Genomic and Synaptic Drivers.* Biol Psychiatry, 2020.

10. Krashes, M.J., et al., *Rapid, reversible activation of AgRP neurons drives feeding behavior in mice.* J Clin Invest, 2011. **121**(4): p. 1424-8.

11. Tsunemoto, R., et al., *Diverse reprogramming codes for neuronal identity.* Nature, 2018. **557**(7705): p. 375-380.

12. Brewer, G.J. and J.R. Torricelli, *Isolation and culture of adult neurons and neurospheres.* Nat Protoc, 2007. **2**(6): p. 1490-8.

13. Martin, M., *Cutadapt removes adapter sequences from high-throughput sequencing reads.*

14. Dobin, A., Davis, C.A., Schlesinger, F., Drenkow, J., Zaleski, C., Jha, S., Batut, P., Chaisson, M., Gingeras, T.R., *STAR: ultrafast universal RNA-seq aligner* Bioinformatics, 2012. **29**(1): p. 15-21.

15. Anders, S., Pyl, P.T., Huber, W.,, *HTSeq—a Python framework to work with high-throughput sequencing data.* Bioinformatics, 2015. **31**(2): p. 166-169.

16. Love, M.I., Huber, W., and Anders, S., *Moderated estimation of fold change and dispersion for RNA-seq data with DESeq2.* Genome Biology, 2014. **15**.
